# Seasonal Changes in Bacterial and Archaeal Gene Expression Patterns across Salinity Gradients in the Columbia River Coastal Margin

**DOI:** 10.1371/journal.pone.0013312

**Published:** 2010-10-13

**Authors:** Maria W. Smith, Lydie Herfort, Kaitlin Tyrol, Dominic Suciu, Victoria Campbell, Byron C. Crump, Tawnya D. Peterson, Peter Zuber, Antonio M. Baptista, Holly M. Simon

**Affiliations:** 1 Division of Environmental & Biomolecular Systems, Center for Coastal Margin Observation & Prediction, Oregon Health & Science University, Beaverton, Oregon, United States of America; 2 CombiMatrix Corporation, Mukilteo, Washington, United States of America; 3 Horn Point Laboratory, University of Maryland Center for Environmental Science, Cambridge, Maryland, United States of America; Argonne National Laboratory, United States of America

## Abstract

Through their metabolic activities, microbial populations mediate the impact of high gradient regions on ecological function and productivity of the highly dynamic Columbia River coastal margin (CRCM). A 2226-probe oligonucleotide DNA microarray was developed to investigate expression patterns for microbial genes involved in nitrogen and carbon metabolism in the CRCM. Initial experiments with the environmental microarrays were directed toward validation of the platform and yielded high reproducibility in multiple tests. Bioinformatic and experimental validation also indicated that >85% of the microarray probes were specific for their corresponding target genes and for a few homologs within the same microbial family. The validated probe set was used to query gene expression responses by microbial assemblages to environmental variability. Sixty-four samples from the river, estuary, plume, and adjacent ocean were collected in different seasons and analyzed to correlate the measured variability in chemical, physical and biological water parameters to differences in global gene expression profiles. The method produced robust seasonal profiles corresponding to pre-freshet spring (April) and late summer (August). Overall relative gene expression was high in both seasons and was consistent with high microbial abundance measured by total RNA, heterotrophic bacterial production, and chlorophyll *a*. Both seasonal patterns involved large numbers of genes that were highly expressed relative to background, yet each produced very different gene expression profiles. April patterns revealed high differential gene expression in the coastal margin samples (estuary, plume and adjacent ocean) relative to freshwater, while little differential gene expression was observed along the river-to-ocean transition in August. Microbial gene expression profiles appeared to relate, in part, to seasonal differences in nutrient availability and potential resource competition. Furthermore, our results suggest that highly-active particle-attached microbiota in the Columbia River water column may perform dissimilatory nitrate reduction (both dentrification and DNRA) within anoxic particle microniches.

## Introduction

The Columbia River and its tributaries, with a drainage basin of 660,480 km^2^, represent the second-largest freshwater discharge in the United States [1], [2]. Due largely to snow melt in the spring, discharge fluctuates seasonally from the highest volumes in April through June to the lowest in September and October. The Columbia River has a profound influence on biogeochemical processes in the coastal ocean through the delivery of nutrients in a massive plume that during times of high discharge reaches dozens to hundreds of kilometers from the river mouth [2]–[4]. We refer in this paper to the Columbia River coastal margin (CRCM) as the continuum between the river, the estuary, the plume and the host continental shelf of the Eastern North Pacific Ocean.

The Columbia River estuary is characterized by a strong tidal cycle, high turbidity, and vertical stratification varying in strength with the tides and river discharge [4]–[7]. Estuarine waters contain biological and chemical gradients established by the mixing of freshwater and seawater [4], [8] that are thought to deeply influence the composition of natural bacterioplankton communities [9]. A productive detrital food web is driven by heterotrophic bacterioplankton, and is based on allochthonous organic material and freshwater phytoplankton that develop seasonally in river impoundments [10]. Tidally-driven estuarine turbidity maxima (ETM) events trap and re-suspend both mineral and organic particles transported through the estuary and extend their residence time several-fold [8], [11]–[13]. As a result, particle-attached bacteria trapped in the estuary by ETM account for approximately 90% of the heterotrophic bacterial activity in the water column [13]. Thus, microbial assemblages are influenced not only by water chemistry, which varies temporally as a function of season, oceanographic conditions, tidal phase and river discharge [4], but also by transport from both the river and adjacent coastal ocean.

Phylogenetic diversity of the estuarine microbial community has been analyzed for both bacteria [14] and archaea [15] using 16S rRNA gene sequence libraries. The majority of free-living estuarine bacterial sequences were found to be similar to tidal freshwater and adjacent coastal ocean environmental clones, while the particle–attached fraction contained a higher proportion (up to 75%) of uniquely estuarine clones [14]. While this particle-attached fraction is known to be more active than the free-living bacterioplankton [13], nothing is known about their specific metabolic properties.

Recent developments in molecular biological tools permit investigations of metabolic responses to fluctuating conditions without a requirement for microbial cultivation; for example, through analysis of gene expression activity. Improvements in protocols for RNA isolation and in techniques for the removal of ribosomal RNA yielded high-quality enrichments of mRNA from environmental samples in recent studies [16]–[19], and subsequent large-scale sequence analysis revealed highly expressed genes [18]. However, the associated high cost of sequencing still limits the number of samples that can be analyzed in this manner, and, therefore, also the spatial and temporal resolution that can be achieved. Alternatively, microarray-based technologies allow for the analysis of a relatively large number of samples at a fraction of the cost of sequencing. DNA microarrays are, therefore, compelling functional tools for investigating microbial transcriptional activity in the environment [20]–[22]. However, for successful microarray applications, several important issues need to be addressed, including (i) selection of representative sets of annotated functional genes, and (ii) specificity of microarray probe hybridization within the context of a large pool of unknown environmental mRNA [22].

We used DNA oligonucleotide microarrays to assess differential gene expression in bacterial and archaeal populations in response to physical and chemical gradients in the CRCM. Freshwater influx from the Columbia River and coastal upwelling from the continental shelf influence biogeochemical cycles by creating large physical and chemical gradients in the water column [12]. This study was undertaken as a first step toward understanding how microbial populations respond, at the transcriptional level, to such gradients.

For this work we employed the CombiMatrix CustomArray™ format, which, in recent studies, produced high quality gene expression data for a variety of complex clinical and field samples [22]–[27]. Using this system, we designed probes for a set of several thousand genes from bacteria and archaea that were selected from functional annotations in the Integrated Microbial Genomes (IMG) data management system of the U.S. Department of Energy Joint Genome Institute (DOE JGI; [28]). Our results indicate that bacterial and archaeal gene expression in the CRCM varied primarily with seasonality in environmental characteristics. Trends involving light, phytoplankton biomass, proximity to the river mouth, and availability of nitrate, phosphate, and dissolved organic carbon (DOC) were observed in the gene expression profiles. An association of gene expression patterns with habitat (tidal freshwater, estuary, plume, or coastal) was also observed, but could not be accounted for by salinity differences alone. The existence of stable, repeating gene expression patterns over temporal and spatial gradients suggests that expression of microbial genes may vary in predictable ways, and, therefore, may be a useful indicator of environmental change. Overall, our results indicate that the highly dynamic CRCM, which is the focus of an NSF Science and Technology Center for Coastal Margin Observation and Prediction (CMOP), is an ideal testing ground for new tools aimed at elucidating microbial metabolic responses to changing environmental conditions.

## Results and Discussion

### Geochemical characterization of CRCM water samples

**Figure 1:**
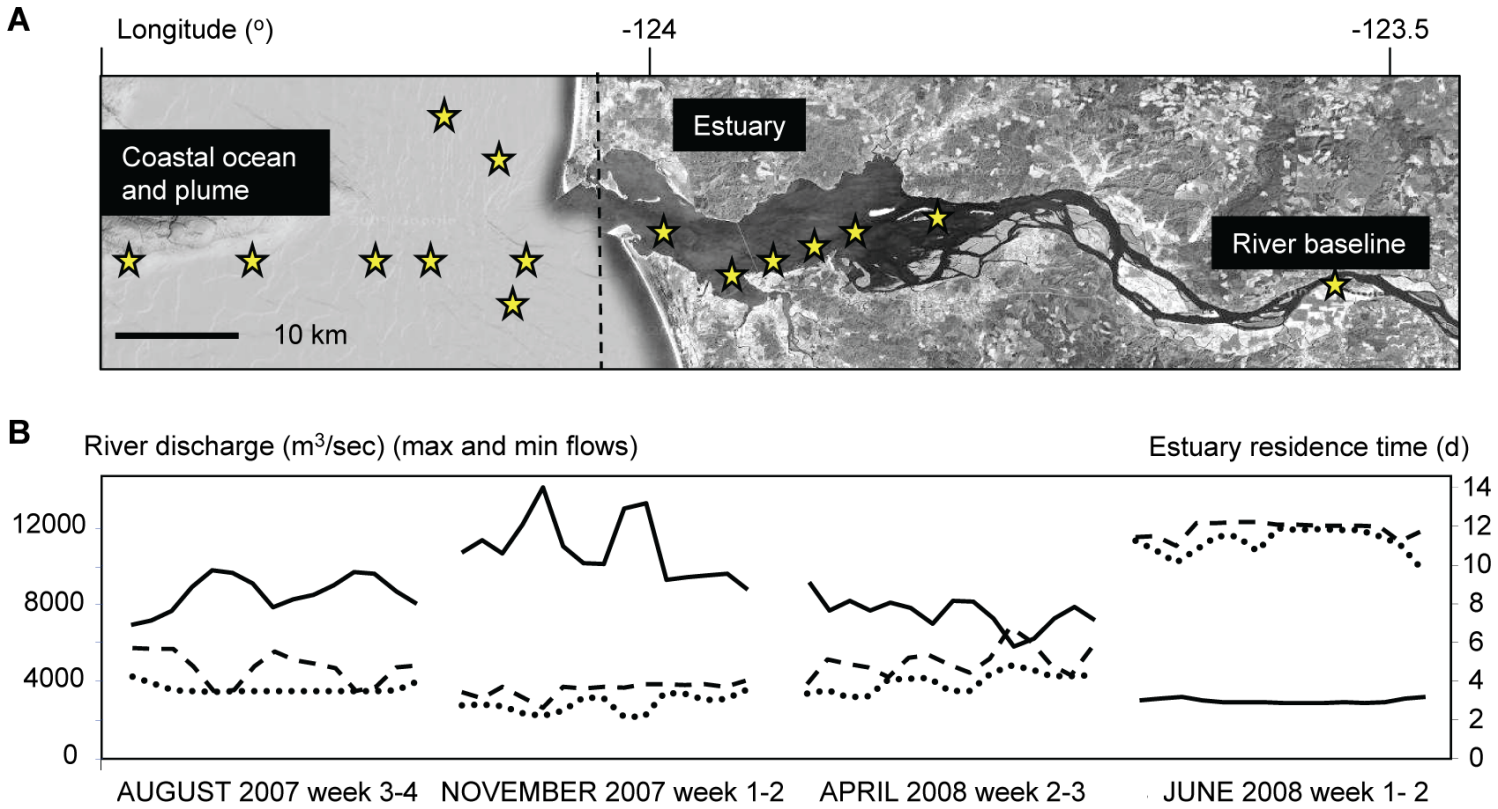
Characterization of the Columbia River coastal margin. (**A**) Sampling locations (indicated as stars) along the CRCM transects. (**B**) Columbia River daily water discharge (dashed and dotted lines indicate minimum and maximum values, respectively), and estuary residence times (solid line), calculated based on U.S. Geologic Survey's National Stream Water Quality Network data collected at Bonneville Dam. Residence times are defined as R = V/(86400*Q), where R is the residence time in days, V is the volume of the estuary in m^3^ relative to mean sea level (defined from Beaver Army to the mouth), and Q is the river discharge in m^3^s^−1^. The data correspond to the 2-week long sample collection times for each month.

**Figure 2:**
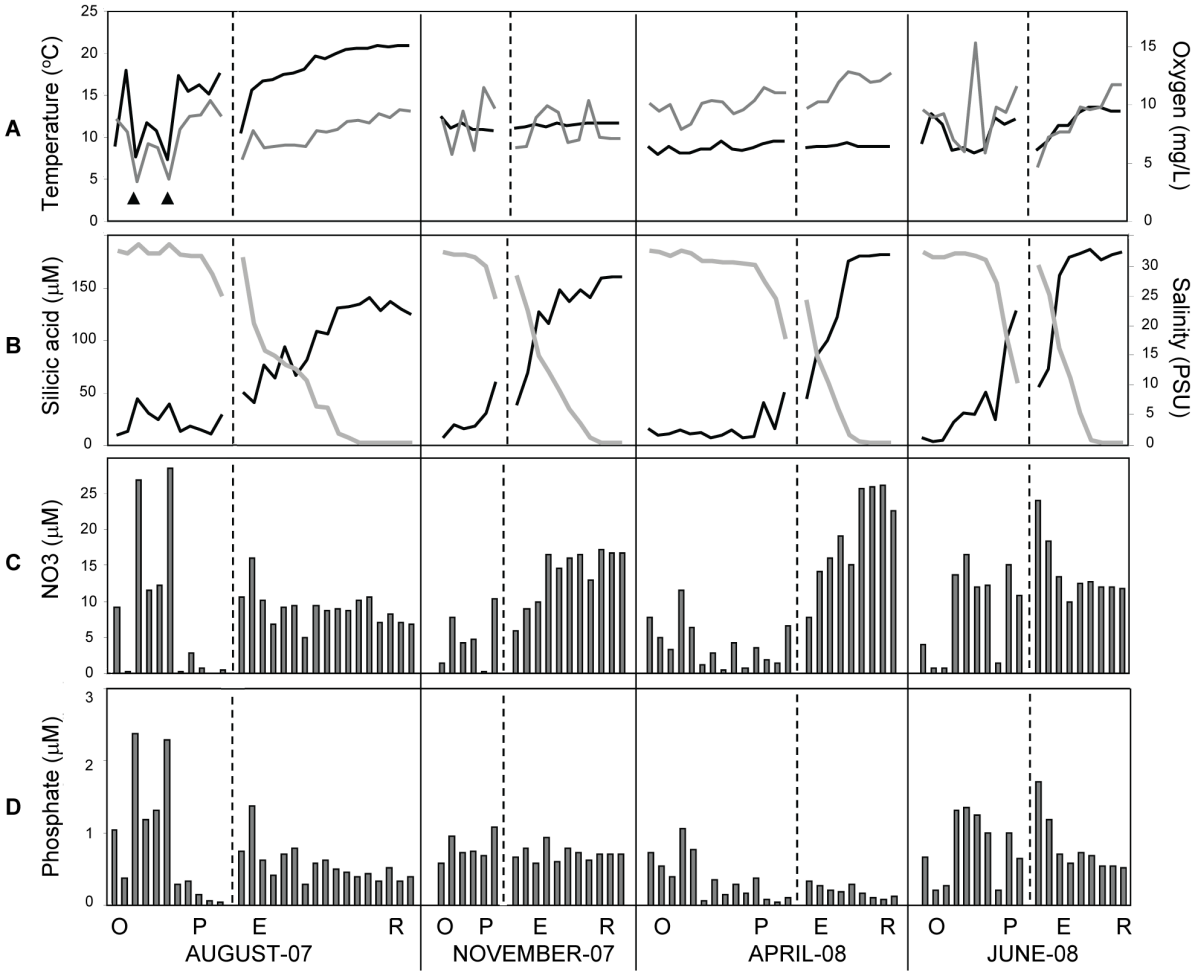
Selected physical and chemical characteristics corresponding to the seasonal sample sets collected for microarray analysis. In all graphs, the X-axis indicates samples sorted first from left to right, according to year and month starting from August and November, 2007, then April and June, 2008; second, within each month by location, starting with the coastal ocean through the plume and estuary to the river; and third, according to salinity within each location, from high (left) to low (right). (**A**) Temperature (black line) and oxygen concentration (gray line); (**B**) Silicic acid concentration (black line) and salinity (gray line); (**C**) and (**D**), nitrate and phosphate concentrations, respectively. Black vertical lines divide seasons, dashed vertical lines within each season divide the ocean (O) and plume (P) from estuary (E) and river (R) samples. The months in which sampling occurred are indicated below.

Our data set consisted of 110 water samples that were collected along transects of the tidal freshwater, estuary, plume and adjacent coastal ocean (Fig. 1A) on ship-based field campaigns. Campaigns were conducted from 7 to 14 days during summer with low river discharge (August 2007), late fall (November 2007), pre-freshet early spring (April 2008), and spring freshet at record high river discharge (June 2008) (Fig 1B). A detailed analysis of the properties of CRCM waters was undertaken to provide environmental context for the gene expression data. Physical and chemical data are plotted in Fig. 2. The X-axis indicates samples sorted (i) from left to right according to year and month starting from August 2007; (ii) then within each month by location, starting with the adjacent ocean through the plume and estuary to the tidal freshwater; and (iii) within each location, according to salinity, from high (left) to low (right). Temperature measurements showed the typical seasonal pattern, with a summer high of 20°C in the river in August, and an average of 8–10°C in spring and fall (Fig. 2A). Oxygen concentrations varied between 7–15 mg/L, and in the river and estuary they were inversely correlated with salinity (*R^2^* of −0.86 in August, and −0.92 in both April and June). Thus, oxygen concentrations were higher in freshwater than in the estuary in all months sampled, except November. (Fig. 2A, 2B). This observation may be explained, in part, by release of oxygen during photosynthesis and growth by freshwater phytoplankton in the river.

### Nutrients

Seasonally-fluctuating discharge volumes in the Columbia River are accompanied by changes in concentrations of macronutrients and organic detritus. Among major rivers, the Columbia River is unusual in being silicic acid rich and nitrate poor during the summer months, with nitrate concentrations of only 2–10 µM in June and July [29], [30]. This is in contrast, for example, to the Mississippi River, in which summertime nitrate concentrations have been measured at >100 µM [31]. Our measurements were consistent with previous monthly analyses of nitrate and silicic acid at the Beaver Army Terminal station (River Mile 53) by the US Geological Survey over the last decade [4]. The silicic acid concentration in the river was high during all seasons and was inversely correlated (*R^2^* = −0.93) with water salinity (Fig. 2B), similar to previous observations [29]. Typical summer nitrate concentrations (∼7 µM) were observed for river water samples collected in August 2007 (Fig. 2C). In winter, nitrate concentrations can be as high as 50 µM, coincident with high winter rainfall and high flow from coastal tributaries [4], [32]. Nitrate concentrations in November 2007 and April 2008 were typical of the ‘shoulder seasons’ before and after the winter nitrate peak, with the nitrate concentrations elevated approximately 3X above the summer levels (Fig. 2C). In April, November and August, the plume and coastal ocean samples had nitrate concentrations that were much lower than those in the tidal freshwater and estuary, consistent with the idea that nitrate flowing into the lower Columbia River from the Columbia basin watershed is typically used within the estuary [2]. Several of the August samples were apparently influenced by upwelling; the two most prominent of these are indicated with arrowheads in Fig. 2A. They displayed clear upwelling characteristics such as reduced water temperature, low oxygen concentration, and highly elevated levels of nitrate and phosphate (Fig. 2C and 2D, respectively). In contrast to the other sampling times, June nitrate levels were similar in the river, plume and a number of ocean samples, consistent with the observation that elevated nitrate concentrations from the watershed may be delivered to the ocean by high riverflow [4]. Finally, ammonium concentrations varied from 1 to 3 µM in the majority of samples, and did not show clear seasonal patterns (Table S1).

Phosphate concentrations in the CRCM also varied seasonally (Fig. 2D), with the highest levels in the adjacent coastal ocean in August, and in the plume and estuary in June. The higher August levels appeared to be at least partly explained by upwelling, while the higher June measurements were likely due to elevated river discharge. A similar dynamic was described for the Tillamook Bay estuary (Oregon, USA), for which elevated estuarine flushing rates produced by high river discharge limited nutrient uptake by phyto- and bacterioplankton, resulting in enhanced nutrient delivery to the coastal ocean [32]. On the other hand, reduced phosphate concentrations were observed in the tidal freshwater and estuary in April with the average N∶P ratio of 131∶1. In late April to early May of 2002 a similar observation was made in the upper portions of the Yaquina Bay estuary, with the N∶P ratio reaching as high as 176∶1 [33]. We speculate that the apparent phosphate depletion might have been caused by an abundant phytoplankton community in the Columbia River in April (see below) under conditions of relatively high nitrate [34], low turbidity and high light. In total, the observed macronutrient dynamics were consistent with previous observations indicating that most nutrients from local watersheds are consumed within the estuary during summer months, rather than exported to the continental shelf [35].

### Microbial abundance and production rates in the CRCM

**Figure 3:**
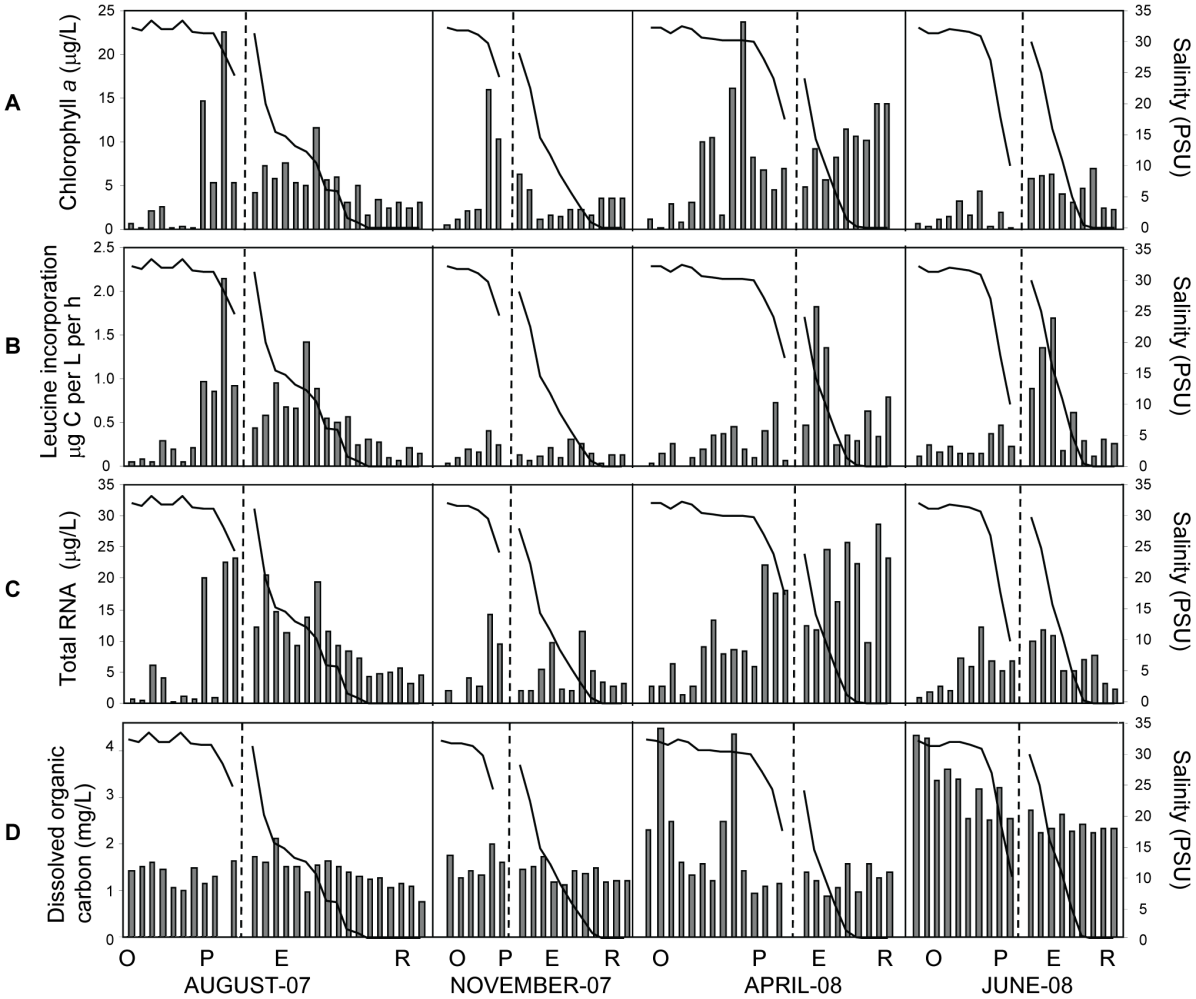
Biological characteristics of the seasonal sample sets collected for microarray analysis. In all graphs, the X-axis shows samples sorted as described in Fig. 2. Salinity plots are superimposed with the bar graphs. (**A**), chlorophyll *a* content; (**B**) bacterial carbon production measured by leucine incorporation; (**C**) total RNA concentrations normalized per liter of sampled water; and (**D**), dissolved organic carbon content. Black vertical lines divide seasons, dashed vertical lines within each season divide the ocean (O) and plume (P) from estuary (E) and river (R) samples. Seasons are shown as months below the graphs.

We measured autotrophic standing stocks (chlorophyll [chl] *a* concentrations), growth rates of heterotrophic plankton (production rates), and total RNA concentrations (total living microbial biomass) in the CRCM samples. The highest correlations among chl *a* concentration, production rate and total RNA concentration were observed in August, with the highest values for all three biological characteristics observed in the estuary and plume (*R^2^* from 0.8 to 0.94), whereas ocean and freshwater end-members had relatively low values (*R^2^* from 0.7 to 0.8) (Fig. 3A–C). Similar, but less pronounced trends for microbial abundance were also observed in June and November in the estuary and plume. In contrast to other sampling times, pre-freshet April samples also showed very high chl *a* and RNA concentrations in the tidal freshwater and at low salinities (0–5 PSU) in the estuary. High RNA and chl *a* concentrations may be at least partly explained by phytoplankton blooms developing in the river freshwater, which is a common occurrence in spring [32], [36]–[38]. This freshwater phytoplankton is believed to perish in the estuary at high salinities, providing detritus for bacterial community development [10]. However, the pheophytin *a* concentration was low (Table S1), indicating that the majority of chl *a* corresponded to living, rather than detrital, phytoplankton biomass. This observation is consistent with observations from Tillamook Bay indicating phytoplankton biomass accumulation in the middle and lower estuary was especially high in spring and summer [32]. Heterotrophic plankton production rates in the estuary were similar in April and June (0.7 and 0.64 µg CL^−1^h^−1^, respectively), and were consistent with previous measurements indicating that heterotrophic activity in the estuary was higher than that in the adjacent coastal ocean or in the tidal freshwater at that time [14], [39].

DOC concentrations were higher in June (4–4.5 mg/L) than in the other seasons (1.5–2 mg/L, Fig. 3D). Measurements of particulate organic carbon (POC) performed on a subset of samples from April, June, and November cruises showed that April POC values in the tidal freshwater and estuary (700 to >1000 µg/L) were almost 5 times higher than concentrations observed in the ocean samples collected in April (235 µg/L) and in November river (tidal freshwater and estuary) water samples (281 µg/L). POC concentrations in the tidal freshwater and estuary in June were also relatively high (670 µg/L).

Given these data, the relatively low microbial abundance (estimated from total RNA concentrations) observed in June compared to April (7 versus 20 µg/L of total RNA; Fig. 3) was unexpected, since macronutrient concentrations in both the estuary and plume were high (Fig. 2C and 2D). However, the June sample collection occurred during the peak of spring freshet at record-high river discharge (almost 3X higher than other sampling times) and under a considerably reduced water residence time of 2 days (Fig. 1B). This suggests that microbial populations require longer water residence times to fully develop in the estuary, even when high nutrient concentrations are present. Chl *a* production was likely also depressed in June due to high turbidity and subsequent low light levels that accompanied the high riverflow. Consistent with this hypothesis, the short water residence times during freshet were believed to prevent the development of the abundant estuarine microbiota typically observed during summer and fall within the Tillamook Bay [32], and in East Coast estuaries (Parker River estuary and Plum Island Sound) [39].

Prior to the onset of seasonal upwelling, the plume tends to have a more freshwater character compared to upwelling-influenced plumes that entrain high-nutrient, high-salinity waters [4]. Consistent with this character and with the patterns observed in the river, chl *a* and total RNA were higher in the plume in April compared to June, despite favorable temperatures, summer light conditions, and abundant macronutrient concentrations in the latter month (Fig. 2C and D, Fig. 3D). We hypothesize that an abundant estuarine microbial community is needed for the development of active plankton in the plume, particularly for the large spring plume, which is characterized by low salinities (Fig. 2B). Plume salinities in June were as low as 10 PSU, in contrast to other seasons, when the plume had salinities of at least 18 to 20 PSU. Alternatively, higher salinities in the plume may also be important for microbial populations to develop, e.g., from an allochthonous marine inoculum.

### Design and validation of functional probes for analysis of gene expression

Oligonucleotide probes for microarray analysis of gene expression were designed with CombiMatrix probe design software (Probe Weaver) based on: (i) uniqueness within a defined gene and sequence set; (ii) Tm and length within a specified range; 70–75°C, and 35–40-mer, respectively; and (iii) absence of stable secondary structures and repeat sequences. Several published studies describe evaluation of the sensitivity and specificity of various CombiMatrix microarray probe sets [23], [25], [26], [40]–[42]. These studies indicated that CombiMatrix probe performance was correlated with theoretical calculations for DNA-DNA hybridizations predicting 1°C reduction in T_m_ for every 1% sequence mismatch [43]–[45]. Theoretically, 3 mismatches in a 35-nt sequence (8.5% sequence mismatch), will result in an 8.5°C drop in T_m_. Empirical validation using the recommended hybridization conditions showed that 3 mismatches reduced T_m_ by at least 15°C, resulting in hybridization signals that were at or below background levels (data not shown). Thus, selection of probes for uniqueness within a specified sequence set by the Probe Weaver software requires at least 15°C difference in T_m_ to the closest hit/s for nontarget sequences in the set.

Little sequence information was available for bacterial and archaeal populations in the CRCM from which to design functional probes; the only relevant data corresponded to 16S rDNA clone libraries constructed by Byron Crump and colleagues [13], [14]. Many of these 16S rRNA gene sequences corresponded to uncultivated organisms about which essentially nothing else was known. Thus, our initial strategy was to design microarray probes with somewhat broad specificity (‘common’ probes) that targeted well-annotated functional genes from sequenced genomes of multiple genera within a phylum. We reasoned that such probes would also recognize homologous genes from uncharacterized microorganisms of the same phylum that were likely to be present in environmental water samples. To evaluate this approach, the CombiMatrix probe design algorithm was applied for probe selection from all predicted open reading frames of a subset of 11 fully sequenced genomes from cultured isolates, including three Alphaproteobacteria (*Pelagibacter ubique*, *Erythrobacter litoralis*, *Roseobacter denitrificans*), three Betaproteobacteria (*Polaromonas naphthalenivorans*, *Polynucleobacter* sp, *Rhodoferax ferrireducens*), one Bacteroidetes (*Flavobacterium johnsoniae*), three Gammaproteobacteria (*Marinomonas* sp., *Marinobacter aquaeolei*, *Nitrosococcus oceani*), and one Actinobacteria (*Salinispora arenicola*).

In total 27,143 non-overlapping 35-mer probes with T_m_ between 65°C and 75°C were designed for the 11 genomes, and then each probe was used in a BLAST search through all genomes using CombiMatrix Genotyper software [23], [41]. In order to identify common probes targeting homologs from several genomes, Genotyper calculated the number of cross-hybridizing sequence hits with less than 15°C difference in T_m_ for each designed probe. Unexpectedly, 80% (21,747) of all probes evaluated produced only one hit: a match of perfect sequence identity from the corresponding genome. Fifteen percent of the probes (4,047) produced 2 hits, the perfect sequence match and one additional partially complementary match from a different genome. Phylogenetic information is limited to the phylum level in Genotyper, thus for the 15% of probes producing more than one hit, two-thirds (2,825) produced a second hit within the same microbial phylum. Finally, 1,349 (5%) of the probes produced 3 or more hits. These probes corresponded to either highly conserved housekeeping genes (i.e. encoding ribosomal proteins), or to genes potentially involved in horizontal gene transfer events (many of these were prophage and transposon-related). Thus, greater than 90% of the microarray probes designed with standard criteria for T_m_ and sequence complexity appeared to be specific for their corresponding target gene and a few homologs within the same phylum, even though they were not originally selected for uniqueness within a large multi-genome context. Although the Genotyper analysis was somewhat inconclusive due to the limited annotations produced by the software, it suggested that sequence diversity was too high to allow design of functional probes that would detect homologs across multiple genera within individual families. Therefore, we instead selected all annotated microbial genes for each functional category, and then designed and tested unique probes for each gene.

Functional genes of interest were selected using the IMG 2.5 system [28]. Approximately 300 sequenced genomes from the Bacteria and Archaea (251 and 52, respectively), representing 246 species, 161 genera and 96 families were selected from taxonomic groups known to be present in the CRCM and/or in water and soil samples from temperate habitats of the Northern hemisphere. Approximately 5000 genes were selected from these genomes, and the corresponding microarray probes were designed for specificity within the set of genes of interest using the standard CombiMatrix Probe Weaver software as described above. In addition, more than 1000 ribosomal RNA sequences from both prokaryotic and eukaryotic microorganisms present in environmental water and soil samples were included into the design process to avoid cross-reactivity with rRNA. The probe sets were synthesized *in situ* on the surface of CombiMatrix CustomArray™ microarrays, and were tested by hybridization with three cDNA pools prepared from 8–10 RNA samples that were collected at different times from disparate locations across the CRCM. The data were normalized using the standard quantile algorithm, and the background signal value was calculated as the mean intensity of the lowest 5% of all signals. Finally, probes that produced significantly high microarray signals – exceeding the background value at least 3-fold in one or more of the pooled target samples – were selected.

The selected probe set was used to create a custom microarray design of 2226 different oligonucleotide probes based on the CombiMatrix 4X2K CustomArray™ platform.

Because we are interested in microbial transformations of nitrogen in the CRCM, the majority (1638, 73.6%) of probes corresponded to genes involved in nitrogen metabolism. Probes for genes involved in carbon metabolism (451, 20.3%), housekeeping functions (100, 4.5%) and light perception and utilization (37, 1.6%) were also included. Phylogenetic composition of the probe set is shown in Table 1. Approximately 21% of the probes represented the Archaea (both Crenarchaeota and Euryarchaeota), 60% represented various Proteobacteria, and the remaining probes (19%) represented Bacteroidetes/Chlorobi, Firmicutes, Acidobacteria and “other” (miscellaneous), divisions. Probe coverage was limited to taxonomic groups containing at least one fully sequenced genome.

**Table 1:** Phylogenetic composition of the 2226 microarray probe set.

Phylum	All probes	Expressed probes	% of expressed	April	June	August	November
**Euryarchaeota**	**356**	**296**	**83.1**	**0.99**	**0.97**	**0.97**	**0.99**
**Crenarchaeota**	**113**	**100**	**88.5**	**1.09**	**1.15**	**1.15**	**1.08**
Gammaproteobacteria	595	564	94.8	1.65	1.68	1.68	1.67
Alphaproteobacteria	426	418	98.1	2.13	2.11	2.12	2.09
Betaproteobacteria	187	184	98.4	1.6	1.59	1.62	1.61
Deltaproteobacteria	147	145	98.6	2.44	2.45	2.29	2.32
Chlorobi	81	81	100.0	1.99	2.05	2.11	1.94
Firmicutes	76	72	94.7	1.59	1.8	1.67	1.75
Actinobacteria	51	47	92.2	2.41	1.95	2.09	2.12
Bacteroidetes	47	39	83.0	1.27	1.2	1.14	1.29
Planctomycetes	37	36	97.3	1.73	1.93	1.67	1.67
Chloroflexi	28	27	96.4	2.11	2.57	2.5	2.57
Epsilonproteobacteria	27	24	88.9	1.42	1.33	1.31	1.47
Magnetococci	13	13	100.0	2.9	3.91	3.01	3.52
Thermotogae	12	12	100.0	1.44	1.86	1.87	1.57
Verrucomicrobia	10	10	100.0	2.92	2.97	4.64	2.68
Zetaproteobacteria	7	7	100.0	1.95	2.19	1.81	2.09
Aquificae	7	7	100.0	2.15	1.84	1.58	2.17
Acidobacteria	6	6	100.0	2.32	3.07	2.62	2.67

The probes were selected as expressed if corresponding signal intensities exceeded the baseline value (background plus 3X standard deviations) in at least 2 samples. The median expression levels were calculated for probes corresponding to expressed genes for each phylogenetic group, and then represented as fold changes over the median signal intensity of the whole microarray data set. Median expression levels for archaeal divisions are shown in bold.

### Bioinformatic analysis of microarray probe specificity using large microbial and environmental sequence databases

Further assessment of probe specificity was done using large microbial metagenome sequence databases that recently became available for batch BLAST searches through CAMERA, the Community Cyberinfrastructure for Advanced Marine Microbial Ecology Research and Analysis (http://camera.calit2.net/index.php [46]). CAMERA currently serves as a repository for approximately 80 microbial sequencing databases, including (i) metagenome data for oceans, lakes, rivers, hot springs and soils (i.e. [47], [48]); (ii) human and animal microbiome sequencing data; and (iii) fully and partially sequenced microbial genomes deposited at the National Center for Biotechnology Information (NCBI). CAMERA's BLAST tool was used for specificity analyses of all 2226 probes against 9 environmental databases of assembled sequences, as well as “CAMERA's Non-Identical Nucleotide Sequences” database, the largest non-redundant database with 65 million sequences totaling approximately 207 gigabases. We applied the CAMERA BLAST search tool with subsequent selection of resulting hits for potential cross-hybridization with microarray probes, using the following criteria: (i) an alignment length of at least 30 nucleotides with *E* value of less than 0.05%, and (ii) at least 90% identity score for the query-match alignment. These criteria resulted in ≥27 perfectly matched positions for each hit, allowing up to 8 mismatches (≥77% overall sequence identity). Because we wished to evaluate the maximum number of potential cross-hybridizing hits, we intentionally chose a less stringent approach than that used by CombiMatrix [41] or in experimental work that determined the threshold value for probe specificity as 87% sequence identity [21].

The CAMERA databases used for this bioinformatic evaluation and the numbers of corresponding hits are shown in Table 2. Only four databases (shown with asterisks in Table 2) provided annotation for the results, which was taxonomic, rather than functional, in nature. As expected, analysis with the environmental virus and eukaryotic microbial sequence databases produced either zero or very small numbers of cross-hybridizing hits (fewer than 10). Only 3 of our probes produced cross-hybridizing hits from eukaryotic genomes, despite the large amount (over 6 gigabases) of non-redundant eukaryotic coding sequences evaluated. Very small numbers of hits were also observed for the databases from Minnesota farm soil, or from open-ocean and deep-water samples from the geographically distant Hawaii ocean time-series. Only 118 probes from our set of 2226 produced hits in the 7 Gb NCBI environmental samples database, reflecting the fact that the Pacific Northwest coastal margin is poorly represented in metagenomic sequencing projects. In contrast, a relatively small 0.8 Gb Moore Foundation Marine Microbial Genomes database produced hits for 526 probes (24% of the total). Thus, the numbers of hits were apparently independent of the database size, and instead were determined by taxonomic, geographic and habitat relevance. Furthermore, almost all probes, 93 to 98% (2,077 and 2,186, respectively), produced hits in the large integrated databases of assembled prokaryotic sequences (bolded in Table 2).

**Table 2:** Cross-hybridizing sequence hits resulting from bioinformatic evaluation of 2226 microarray probes against the CAMERA repository.

Database name as defined in CAMERA	Total length (bp)	# sequences	# Cross-hyb hits	# probes with hits
GOS: Site-specific 16S Sequences (N)	3,118,182	4,125	n/a	0
GOS: move858 Assembled 0.002-0.22 Chesapeake Bay (N)	8,669,804	5,357	n/a	0
FarmSoil: Assembled Sequences (N) (Minnesota farm soil)	144,897,582	139,340	2	2
HOT: All ORFs (N) (Hawaii Ocean Time-series ALOHA)	169,784,453	449,086	2	1
Moore Foundation Marine Microbial Genomes (N)*	856,811,427	12,886	630	526
GOS: Combined Assembly Coding Sequences (N)	3,668,987,939	6,115,750	137	62
Eukaryotic Microbial Genomes (N)*	6,342,658,807	1,453,409	3	3
All NCBI Environmental Samples (ENV_NT)	7,194,061,284	17,695,887	218	118
All Prokaryotic Genomes (N)*	9,577,197,991	655,666	3770	2,186
CAMERA's Non-Identical Nucleotide Sequences (N)*	179,511,589,666	38,512,986	4,367	2,077

Columns 2 and 3 show the total amount of sequence information and the number of individual sequences, respectively, within each database in Column 1. Column 4 shows the numbers of sequences selected as potentially cross-hybridizing with the microarray probes. Column 5 shows the numbers of microarray probes that produced at least one hit in the corresponding CAMERA databases. Asterisks show databases with taxonomic annotations. The two databases used for taxonomic analysis of probe hits are shown in bold.

The distribution of microarray probe hits within CAMERA's ‘All Prokaryotic Genomes” and ‘Non-Identical Nucleotide Sequence’ databases, the two largest databases from Table 2, were analyzed to determine taxonomic rank, and the results are shown in the Table 3. Only a small number of probes (1.8 to 7%, Table 3) did not produce any significant hits. The majority of probes (86 to 91%) produced BLAST hits from the same genus that was used to design the probe (and typically, also from the same species, although this was not always determinable due to incomplete annotations). When matches to multiple genera within a single microbial family were allowed, 91% to 97% of the probes placed into this category (Table 3). Approximately 1.5–2% (34 to 46, Table 3) of probes hit genes from organisms outside of the microbial family containing the target organism. Among them fewer than 1% hit genes outside of the target organism's phylum (8 to 17 probes, for ‘All Prokaryotic’ and ‘CAMERA's Non-Identical’ databases, respectively). All but one of the probes in this latter group produced multiple hits, which typically corresponded to genes from the same genus/family as the target organism, and 1–2 hits from outside of the family. Taken together, our analyses indicated that the microarray probe set was largely specific for the target gene and organism, but approximately 10 to 15% of the probes also detected homologs from different genera within the corresponding target family, or, in very rare cases, outside of the targeted family. These results, therefore, suggested that the probe set might be useful not only for detecting gene expression from targeted organisms, but also from related, but currently uncharacterized microorganisms in the CRCM.

**Table 3:** Distribution of microarray probe hits within CAMERA's Non-Identical Nucleotide Sequence and All Prokaryotic Genomes databases.

Database	CAMERA's Non-Identical	All Prokaryotic
Total number of probes with hits	2,077 (93.3%)	2,186 (98.2%)
Probes with single hits	1,459 (65.5%)	1,595 (71.6%)
Probes with double hits	330 (14.8%)	339 (15.2%)
Probes with multiple hits	288 (12.9%)	252 (11.3%)
Probes with all hits from the same genus	1, 921 (86.3%)	2,019 (90.7%)
Probes with all hits from the same family	2,031 (91.4%)	2,152 (96.7%)
Probes with some hits outside of the family	46 (2%)	34 (1.5%)

The numbers in brackets are percentages of the total number of probes (2,226).

### Experimental validation of microarray probe specificity

Two approaches were used for experimental validation of microarray probe specificity under the microarray hybridization conditions used in this study. The first was aimed at determining whether the probes cross-hybridized with rRNA-derived targets, which is considered to be a serious problem in microarray applications to environmental samples [26]. For this reason, the probes were designed against cross-hybridization with over 1000 rRNA sequences (both prokaryotic and eukaryotic). In addition, application of the CAMERA Blast tool to evaluate probe matches to the ‘GOS Site-specific 16S Sequences’ database containing over 4,000 assembled sequences (over 3 Mb in total, Table 2), did not produce any significant hits. To evaluate cross-hybridization of rRNA in the target preparations, we performed subtractive hybridization assays. For the majority of probes, the rRNA-depleted samples produced higher signals in comparison with untreated samples (data not shown). Thus, an increased proportion of mRNA in the target preparations resulted in an increase in signal intensity, indicating that our probes hybridized specifically with corresponding mRNA-derived targets (and did not cross-hybridize with rRNA).

Additional validation was performed using laboratory-grown cultures of *Bacillus subtilis* and *Pseudomonas putida*. Total RNA from each culture was isolated, converted to fluorescently labeled cDNA, and hybridized in duplicate to microarrays. A typical example of the raw signal intensity data sorted by organism annotation is shown for microarray hybridization with *B. subtilis* targets (Fig. S1). High signal intensities exceeding background at least 10X were observed for 9 out of 10 of the probes designed from *B. subtilis* functional genes. In contrast, only 3 probes from closely related *Bacillus* species (∼15% of probes in this category) produced similar signals with *B. subtilis* cDNA. For probes corresponding to unrelated species from other families, 0.2% produced signals exceeding 3X background, and 9% produced signals exceeding 1X background. Thus, 99.8% of unrelated probes had signal values that did not exceed 3X background (calculated as the lowest 5% of all signals), which was our cutoff for positive microarray signals. Similar data were obtained for *P. putida* laboratory cultures (not shown). Experimental validation of probe specificity closely mirrored results from the bioinformatics analysis, indicating that the majority of probes were specific for the intended target, while approximately 15% of probes to genes from closely related taxa, and only a very minor proportion (0.2%) of the probes to genes from organisms outside of the targeted taxonomic family, hybridized to *B. subtilis* cDNA.

### Microarray data acquisition from environmental samples and analysis of reproducibility

Total RNA from 64 samples (14 from August, 2007; 17 from November, 2007; 17 from April, 2008; and 16 from June, 2008) was used to prepare microarray hybridization targets by reverse transcription into fluorescently labeled cDNA. Due to relatively high microbial abundance in the CRCM, the RNA yields were sufficient to generate labeled targets without additional RNA amplification steps, thus preventing potential bias introduced by this common technique [22]. The targets were hybridized in duplicate, and replicate hybridizations were compared using scatter plots to estimate experimental variation (Fig. S2). The replicate data were highly consistent when the same target was hybridized to two different sectors of the same microarray chip, to different microarray chips, or to the same microarray chip upon re-use (Fig. S2A). The data points grouped around the 45° line and were well within the standard 2-fold cut-off lines (Fig. S2A). The corresponding correlation coefficients (*R^2^*) were >0.98, demonstrating that inter- and intra-chip variation of the microarray platform was quite low. A similar approach was used to evaluate variability introduced by sampling and target preparation protocols (Fig. S2B). We observed high reproducibility (*R^2^*≥0.96), for target preparations from the same RNA in two independent reverse transcription reactions, or from two different freshwater samples collected at the same location at approximately the same time (Fig. S2B). However, increase in the variation (*R^2^* = 0.94) was observed if two water samples were collected at different river locations 50 km apart on the same day (Fig. S2B, right). Taken together, these data indicate that experimental variability was determined largely by sampling location, and not by sample handling, RNA isolation, or microarray hybridizations. Thus, duplicate hybridizations performed for the same sample were averaged to create mean expression values. The data were deposited according to the MIAME reporting guidelines and are accessible from the Gene Expression Omnibus (GEO) public database (http://www.ncbi.nlm.nih.gov/projects/geo/) with the accession number GSE18303.

### Selection and analysis of significantly expressed genes

Genes were selected as significantly expressed if the corresponding probes produced normalized signal intensities exceeding the background value plus 3X standard deviation in at least 2 different samples. Among 2226 genes represented on the microarrays, 2076 (93%) fit this criterion, and were selected to compare gene expression levels of the major prokaryotic phyla represented on the microarrays (Table 1). The median expression value was calculated for the whole dataset and for each phylum separately in four sample groups, representing different sampling times. Phylum-specific gene expression levels are shown in Table 1 as fold changes relative to the overall median expression value for all normalized microarrays. For bacteria, these levels varied from 1.3 to 4.6 fold (Table 1), with the highest values (approximately 2.5 to 4.5-fold across all seasons) observed for genes from the Verrucomicrobia and unclassified Proteobacteria (in particular, Magnetococcus). Relatively high levels of expression (>2-fold across all seasons) were also observed for genes of Chloroflexi, Acidobacteria, Alphaproteobacteria and Deltaproteobacteria. In contrast, expression levels for both archaeal divisions were close to the median and much lower than for many of the bacterial groups (Table 1). As is true in general for microarray approaches, observed changes in the signal for a particular transcript may be influenced by abundance of the organism expressing the transcript, by differential regulation of the corresponding gene, or both. In any case, among 246 prokaryotic species represented by 2226 microarray probes, 244 had at least one significantly expressed gene in multiple samples, validating our initial selection of genomes.

**Figure 4:**
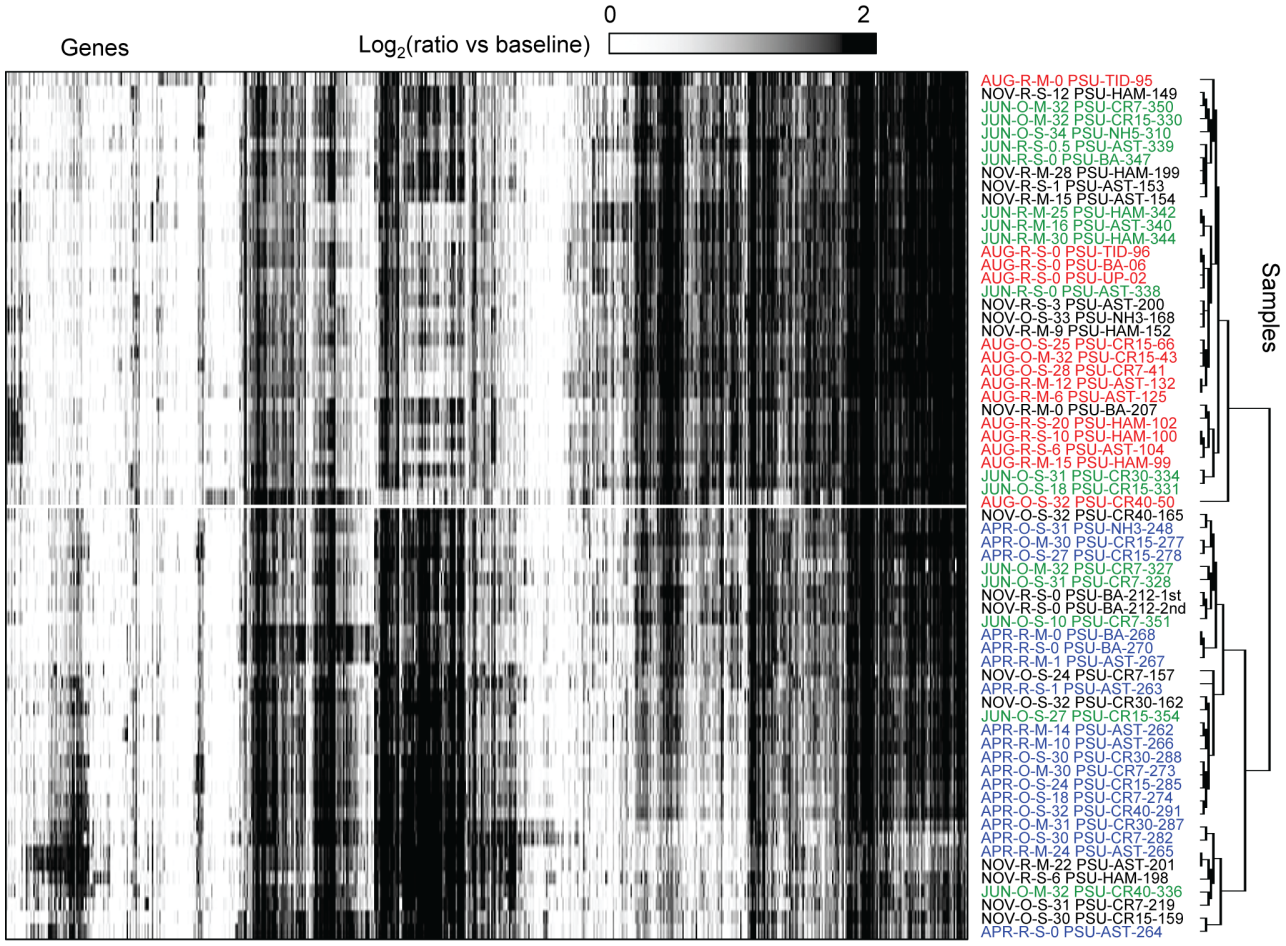
Expression profiles in the seasonal sample sets, displayed as fold changes over the background. Two-dimensional clustering of 2076 probes corresponding to expressed genes (columns) and 56 samples (rows) was done using BRB ArrayTools software based on similarity matrix calculated with an agglomerative algorithm, and complete link correlation. Expression values are colored according to the ratio of signal to the background (displayed using a logarithmic scale). White to black scale indicates magnitude (from low to high). Sample names are composed of season (Apr, Jun, Aug, and Nov), habitat (R, river and estuary; O, plume and coastal ocean), depth (S, surface 1–3 m; M, mid-depth 4–50 m), location, and unique sample number. Location codes: number shows distance from the coast in km; CR, Columbia River transect in the plume and coastal ocean; NH, Newport Hydroline transect in the coastal ocean at Newport; AST and HAM, estuary locations near Astoria (river mile 7–9) and Hammond (river mile 5), respectively; TID, estuary locations in the tidal basin (river mile 22–23); BA, river location at Beaver Army Dock (river mile 53) near Quincy, Oregon; UP, river at mile 74.

For analysis of gene expression patterns in the 64-sample set, the signal value for each probe in each sample was calculated as the log-transformed ratio of normalized intensity versus the background (common for all microarray data after normalization). Two-dimensional (2D) clustering of genes (X-axis) and samples (Y-axis) is shown in Fig. 4. The heat map clearly indicates that the majority of genes were significantly expressed in multiple samples, and changes were observed in the corresponding gene expression profiles with respect to different seasons (described below). These data suggest that similar microbial taxa were metabolically active in different habitats across the CRCM, and were consistent with the fact that the probes most likely detected similar gene expression patterns from different species within a microbial family across sampling sites.

Clustering of samples by similarity of gene expression patterns is shown as a dendrogram on the right side of the heat map, which divides the samples into two major clusters (Fig. 4, upper and lower clusters are divided by a white line). June and November samples were present in both clusters. April and August samples, however, placed into lower and upper clusters, respectively, clearly separating gene expression profiles in April and August from one another. This pronounced seasonal difference in gene expression patterns evidently exceeded the differences in expression across habitats with different salinities (tidal freshwater, estuary, plume and adjacent coastal ocean). Consistent with this observation, calculated pair-wise correlation coefficients (*R* ^2^) for expression patterns in samples with variable salinities collected from the estuary and plume and analyzed for each season were 0.8 to 0.9 (Fig. S3). Correlation to freshwater end-member expression patterns decreased with increasing salinity, and expression patterns differed most in ocean samples at high salinities (*R^2^* from 0.6 to 0.7). This indicates that estuarine microorganisms may carry out similar ecological functions over a range of salinities, and supports the idea put forth by Crump *et al.* [14] that they form communities that are distinct from those in tidal freshwater and the adjacent coastal ocean. Also consistent with the results presented here, a recent report indicated that seasonal differences in the composition of Chesapeake Bay bacterioplankton assemblages were more significant than the variation accounted for by gradients in salinity [49].

### Variation in differential gene expression patterns across salinity gradients in the CRCM with season and habitat

**Figure 5:**
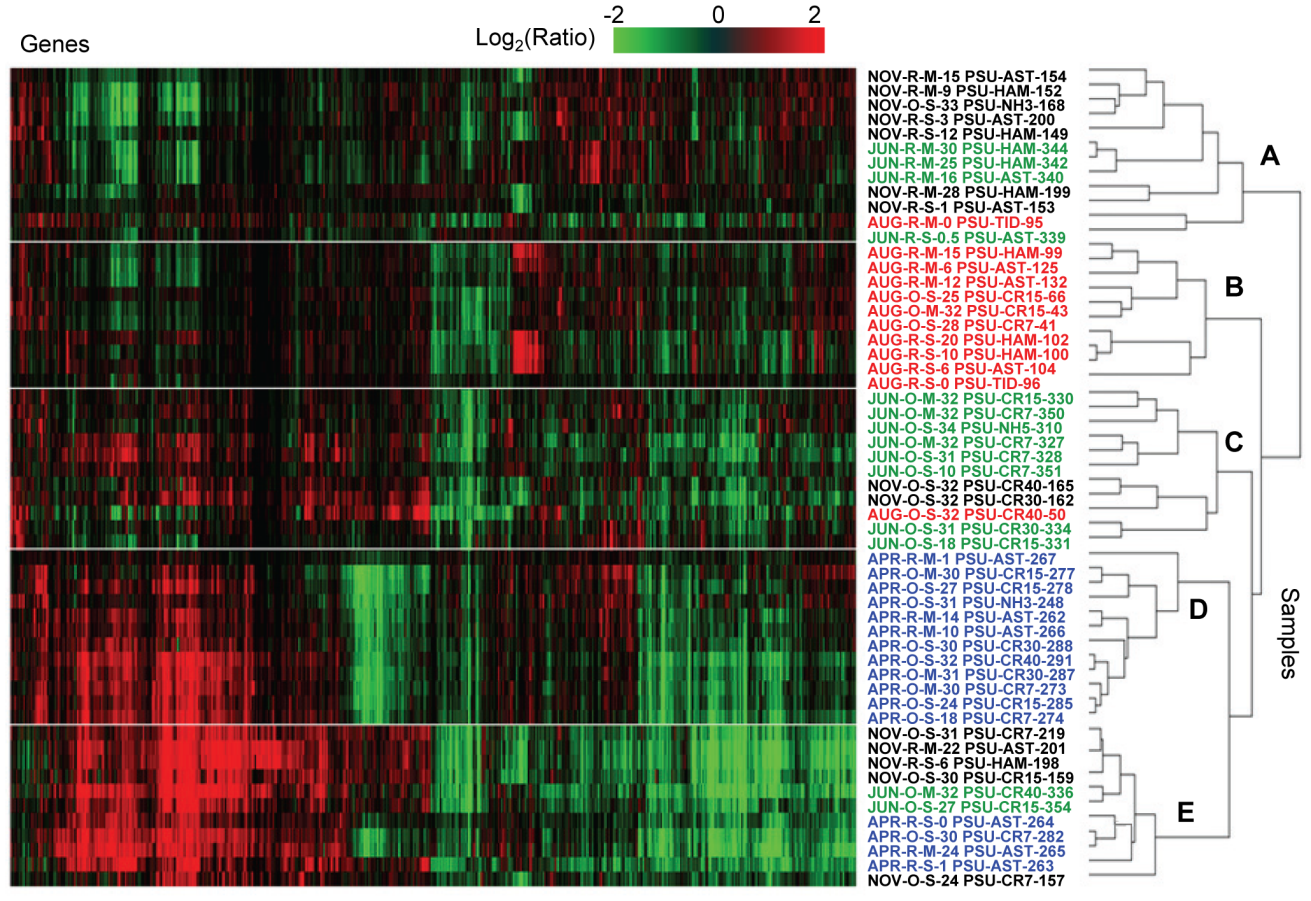
Differential expression across salinity gradients of the CRCM compared to seasonal tidal freshwater baselines. Gene expression ratios were calculated for each probe in each sample as log (base 2) transformed ratios over the corresponding seasonal average freshwater values. Genes were selected as differentially expressed if they had ratios over 2-fold in at least two different samples. Two-dimensional clustering of 1496 differentially expressed genes (columns) and 56 samples (rows) was performed using BRB ArrayTools software based on similarity matrix calculated with an agglomerative algorithm, and complete link correlation. Expression data are colored according to fold changes in the mRNA levels (displayed using a logarithmic scale). Green and red colors indicate decreased and increased values, respectively, within a sample relative to the seasonal freshwater baseline. The color scale indicates the magnitude of change. Sample names are colored according to the season: blue, April 2008; green, June 2008; red, August 2007; and black, November 2007. Samples are designated as described in Fig. 4.

We evaluated season- and habitat-specific differential gene expression in archaeal and bacterial populations in the estuary, plume and adjacent coastal ocean compared to the tidal freshwater baseline. The baselines were calculated for each sampling season by averaging of the two freshwater samples collected at the same river location (0 PSU). For samples collected within a season, gene expression ratios were calculated relative to the corresponding freshwater baseline. Overall, 1496 (72% of the total) genes exhibited greater than 2-fold ratio changes in at least two samples, and these were selected for 2D clustering analysis (Fig. 5). The clustering diagram was dominated by five distinct patterns, designated “clusters A–E”; each consisting of hundreds of expressed genes (Fig. 5, divided by the white lines). The differential gene expression patterns could not be accounted for by one unique environmental factor, or by linear combinations of factors. However, use of Primer 6 software to generate multidimensional scaling plots of maximum variability among samples revealed a seasonal trend in the data set (Fig. S4), similar to results shown in Fig. 4, with April and August samples clustering separately. Also consistent with the previous analysis, MDS plots did not reveal distinct clustering of samples based on habitat (tidal freshwater, estuary, plume or adjacent ocean). Close examination of the 2D clustering diagram in Figure 5, however, suggested additional trends in the dataset. Cluster A was composed of samples from low-light habitats in the estuary, mainly from November. This cluster also included estuarine samples that were collected from below the surface in June (4) and August (1), and one sample collected at the surface in June when turbidity was extremely high (data not shown). The relatively low differential gene expression observed in this cluster compared to the freshwater baseline samples can be explained by the fact that the river also experienced low light levels in November, as well as high turbidity in June. Photosynthetically active radiation (PAR) data were collected only for June and April, but the values corresponding to samples collected at 2-m depth in the estuary were almost 3X higher in April than in June (2.7×10^13^ versus 9.5×10^12^ micromole/m^2^/s, respectively). Consistent with the idea that light levels may have influenced sample clustering, analysis of the small set of probes on our microarrays corresponding to genes involved in light perception and utilization indicated that many of them produced relatively low signals for the samples within cluster A, and for June and November baseline samples, while the corresponding microarray probe signals were 2–10X higher for April samples in clusters D and E.

Cluster B was composed of samples collected entirely in August across habitats characterized by variable salinities. Microbial biomass was dominated by phytoplankton throughout the CRCM in August (Fig. 3A and C), which could account for the similarities in gene expression patterns compared to the freshwater baseline. In fact, the two August samples that did not fall into this cluster contained the lowest concentrations of chl *a* (Table S1). Also consistent with this idea, a large bloom of the ciliated protist *Myrionecta rubra*, containing cryptophyte chloroplasts, was observed in the estuary during the August cruise in 2007. Samples in cluster B were also characterized by higher temperatures compared to those in other groups, but because this was also the case for the freshwater baseline samples, increased temperature alone would not have resulted in the differential gene expression patterns that were observed.

Clusters C–E contained samples exhibiting the highest levels of differential gene expression. Cluster C was composed of samples collected at high salinities in the plume and adjacent coastal ocean in June, at locations where chl *a* and total microbial abundance (total RNA) were very low (Fig. 2) and DOC was high (Fig. 3). This was in contrast to the June estuary samples, which did not fall into this cluster, and the freshwater baseline samples, both of which had higher concentrations of chl *a* and lower DOC. One August and two November ocean samples included in cluster C also had low total RNA and chl *a* concentrations (Fig. 2), and intermediate DOC levels (Fig. 3). Thus, both salinity and availability of DOC may have influenced the grouping of samples into this cluster.

Clusters D and E contained samples from seasons when nitrate levels were high in the tidal freshwater baseline. The samples had nitrate concentrations that were, on average, 25% (cluster D) and 47% (cluster E) of the concentrations in corresponding seasonal freshwater baseline samples (Table S1), suggesting nitrate uptake by active microorganisms. Nitrate concentrations in cluster C samples were also about 70%, on average, of the levels in the corresponding freshwater baseline samples. This interpretation is consistent with the relatively high levels of differential gene expression observed for samples in these three clusters. For cluster D (April) samples, the nitrate:phosphate ratios were additionally 4–20X greater in the river baseline samples compared to the estuary and plume (240 versus 59 and 11, respectively, Table S1 and Fig. 3), which may correspond to the grouping of samples into this cluster.

Cluster E was composed of samples collected near the mouth of the Columbia River (with one exception) in November, June, and April. Close proximity of cluster E samples to the mouth of the river, and the failure to determine trends from other measured variables suggests that gene expression patterns may be correlated with factor(s) that we did not measure. All together, the trends described above suggest that bacterial and archaeal gene expression in the CRCM may be influenced (either directly or indirectly) by light (clusters A, D, E), phytoplankton biomass (cluster B), salinity (cluster C), proximity to the river mouth (cluster E), and availability of DOC (cluster C), nitrate, and phosphate (clusters C, D, E).

### Stability in gene expression patterns over time and across salinity gradients

Within each cluster, the highest similarity in gene expression patterns was observed for samples collected close together in space (in the estuary and plume) and time (1–2 day intervals). This was the case even though water salinity in these samples varied widely, e.g. from 10 to 30 PSU in April (Fig. 5, cluster D) and 6 to 25 PSU in August (Fig. 5, cluster B). Some of these samples had nearly identical gene expression patterns, despite being collected at different stages of the tidal cycle. With the exception of salinity, the environmental data for samples with such highly similar gene expression patterns were also very similar, with calculated pair-wise correlation coefficients in the range of 0.8–0.85 (for all numeric environmental data in Table S1). In November and August, some samples collected 5 to 10 days apart were also highly similar in terms of gene expression patterns, and, again, the temporal stability of these patterns corresponded to relatively similar environmental conditions (*R^2^* from 0.7 to 0.75). This suggests that gene expression, and, potentially, metabolic activity of microbial assemblages in the estuary and plume are stable over time and across a wide range of salinities, as long as other physical and chemical factors remain relatively unchanged.

### Functional gene expression signatures associated with salinity gradients of the CRCM

The largest and most uniform common gene expression pattern encompassed all of the April samples and many November and June samples, and it is represented by clusters C, D, and E (Fig. 5). Self-organizing maps (SOM) and Analysis of Variation (ANOVA at *P* values below 0.001) were used to select probes pertaining to this prominent expression pattern in the corresponding samples. The results of the two analyses were nearly identical, and included approximately 500 probes with higher relative signals compared to the freshwater baseline.

**Figure 6:**
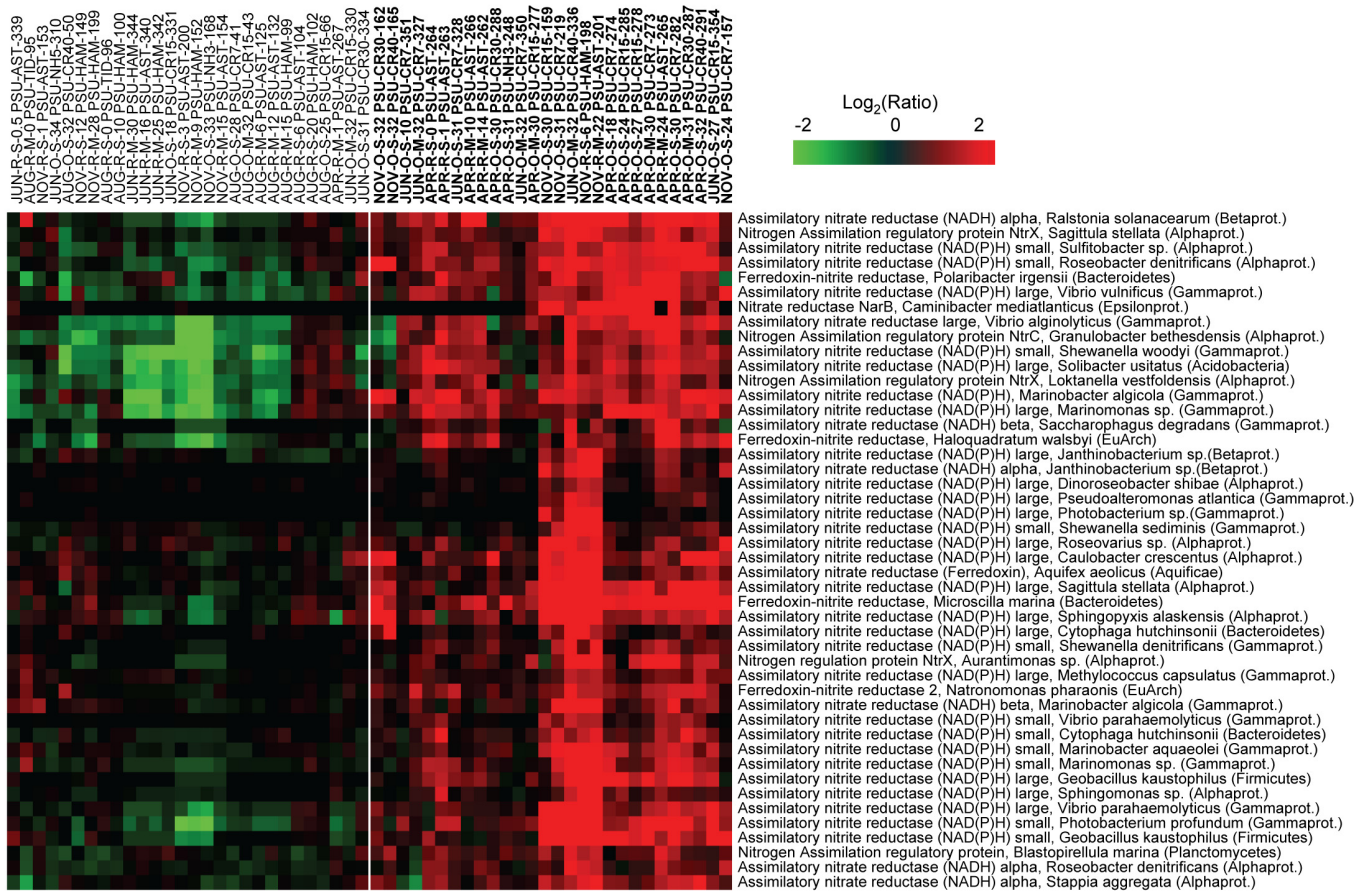
High relative expression of nitrate assimilation genes in comparison to the corresponding seasonal freshwater baseline. Gene sets were selected from clusters C–E of Fig. 5. Expression ratio calculations, 2D clustering, and the heat map display were prepared as described in Fig. 5, except that samples are shown as columns, and genes as rows. Gene names include the corresponding organism and phylum information. The sample cluster that has the upregulation pattern is separated from the rest of heat map by the while line, and the corresponding sample names are shown in bold.

**Figure 7:**
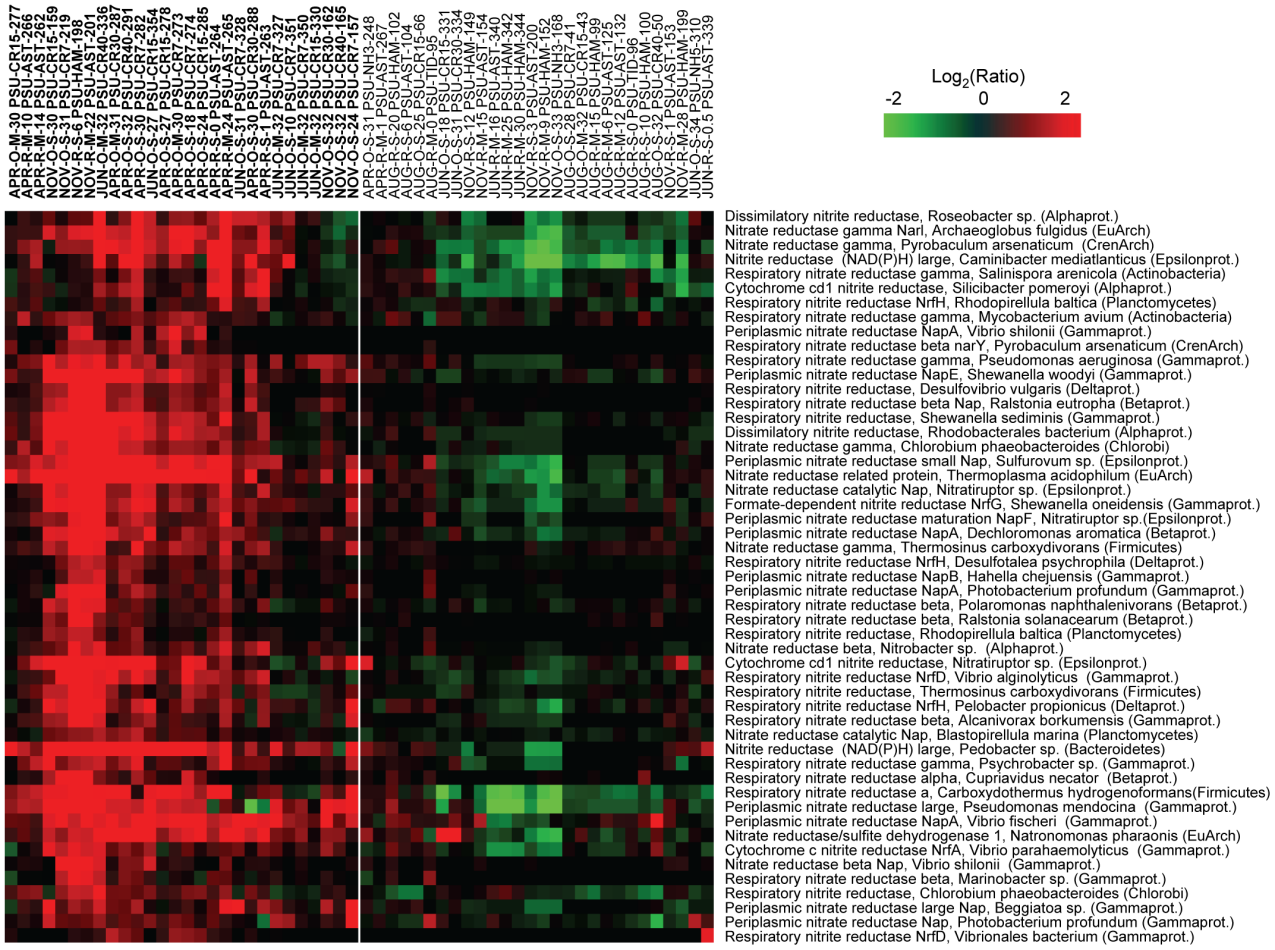
High relative expression of denitrification pathway genes in comparison to the corresponding seasonal freshwater baseline. Gene sets were selected from clusters C–E of Fig. 5. Expression ratio calculations, 2D clustering, and heat map display were done as described in Fig. 5 and 6.

**Figure 8:**
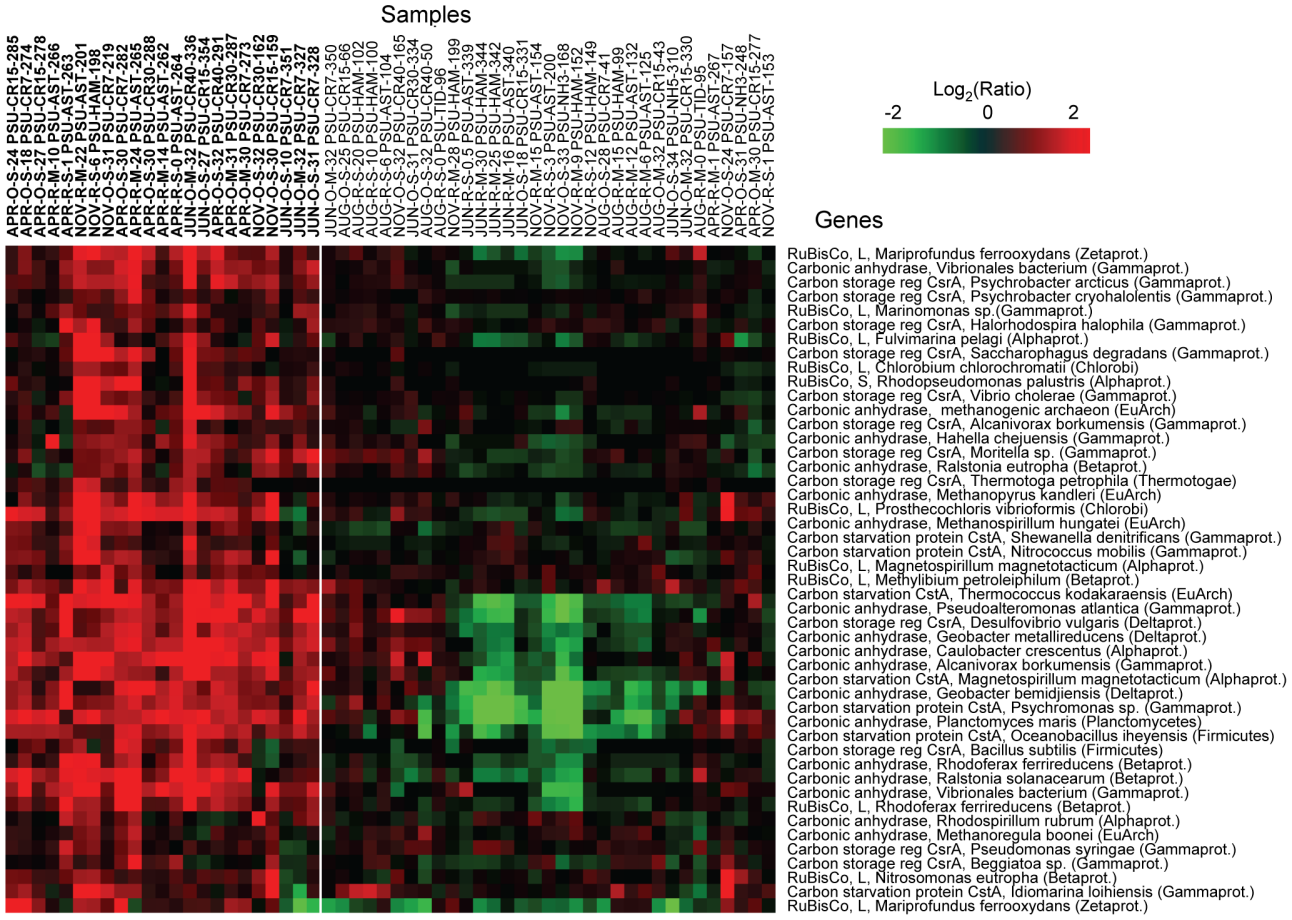
High relative expression of genes involved in carbon fixation, scavenging, and storage. Gene sets were selected from clusters C–E of Fig. 5. Expression ratio calculations were performed relative to the corresponding seasonal freshwater baseline; 2D clustering, and heat map display were done as described in Fig. 5 and 6.

Several functional gene groups were represented by the probe subset, including genes involved in nitrate assimilation (Fig. 6), dissimilatory nitrate/nitrite reduction (e.g., in denitrification and dissimilatory nitrate reduction to ammonia pathways, Fig. 7), and carbon utilization pathways (Fig. 8). The corresponding 143 probes used to generate the data for Fig. 6, 7, 8 were additionally evaluated by BLAST searches against the entire NCBI nucleotide sequence database using the criteria described above. The majority of probes (138 out of 143) produced hits only to the intended functional gene target within the same microbial genus. Five probes out of 143 produced an additional hit from a different genus within the same family. These results were consistent with the bioinformatic and experimental probe validations described above, and indicated that the selected probe subset hybridized with specific functional targets >96% of the time.

The complete probe set used for microarray analysis contained all annotated genes for nitrogen metabolism for each included genome, and in many cases, genes from both nitrate assimilation and dissimilatory nitrate/nitrite reduction pathways were present in the same organism. However, the phylogenetic composition of the two associated probe subsets showed very little overlap (less than 15%), indicating that the enhanced signals for genes in these pathways likely corresponded to their expression in different organisms. The relatively high expression observed for both of these gene sets appeared to correspond to lower nitrate concentrations in the estuary, plume and adjacent coastal ocean compared to the freshwater baseline in April, November and June (discussed above) and to the (inferred) increase in nitrate uptake. Similar to our results, Glibert *et al.* [50] also found that nitrate uptake was relatively higher in April in the plume of the Chesapeake Bay estuary compared to other months during late spring, summer and winter.

Although both denitrification and DNRA are prominent processes in marine and estuarine sediments, expression of the genes involved in these pathways in our water samples was surprising because low levels of oxygen are required for cells to respire nitrate, and oxygen concentrations in the CRCM were relatively high during all analyzed seasons (Fig. 2A). We therefore hypothesize that denitrification and DNRA may be carried out by particle-attached bacteria that reside within suspended sediment particles, where oxygen may be limiting. Assuming microarray signals relate directly to enzymatic activity, our working hypothesis is that particle-attached organisms, which encounter low-oxygen microenvironments, expressed the genes for dissimilatory nitrate reduction in the water column, while free-living, or both particle-attached and free-living microorganisms, expressed nitrate assimilation genes. Alternatively, high expression of DNRA and denitrification genes may have resulted from residual activity of organisms transported from sub-oxic habitats, including estuarine sediments or the adjacent hyporheic zone. However, denitrifiers, at least, are often facultative anaerobes that do not require strictly anoxic conditions for respiration of nitrate [51], and the isolation of bacterial strains capable of denitrification under aerobic conditions has been reported [52]–[54]. Future biogeochemical rate measurements are needed to determine if these processes are occurring on suspended particulate matter in the Columbia River water column, a phenomenon that has been observed in the River Rhone plume and coastal waters of the northwestern Mediterranean Sea [55], [56].

Another probe subset with elevated signals corresponded to differential expression of genes involved in carbon utilization pathways (Fig. 8). These genes were relatively highly expressed in 13 out of 16 April samples, and also in 5 ocean samples from June. The gene group included carbon fixation genes encoding ribulose-1,5-bisphosphate carboxylase oxygenase (RuBisCO), the enzyme used to catalyze the first major step of carbon fixation in the Calvin cycle, and carbonic anhydrase (CA), the key enzyme of the CO_2_ concentrating mechanism [57]. Based on the relatively high expression observed in April samples of such genes involved in prokaryotic primary production, it appears that blooms of autotrophic prokaryotes may occur in the CRCM in spring, similar to the eukaryotic phytoplankton blooms that occur there at that time [10]. Consistent with this idea, a recent study showed that a spring phytoplankton bloom in the Sargasso Sea was accompanied by a bloom of Alphaproteobacteria [58].

In addition, the highly expressed probe subset contained genes for the carbon starvation protein, CstA, and for the carbon storage regulator protein, CsrA. Both are key members of the global carbon storage regulatory (Csr) system involved in nutrient scavenging, utilization of alternative carbon sources, and quorum sensing [59]. Expression of the *cstA* gene, shown to encode a peptide transporter in *Escherichia coli* [60], is consistent with reports that dissolved combined amino acids (DCAA) are components of estuarine and marine dissolved organic matter and are utilized as a source of carbon and nitrogen by heterotrophic bacteria [61]. Because coverage of genes involved in carbon metabolism was limited to a few groups in the current probe set, future iterations will include genes for more pathways, with the goal of examining the relationship between microbial gene expression and dissolved organic matter utilization in the CRCM.

### Phylogenetic analysis of differential gene expression patterns

**Figure 9:**
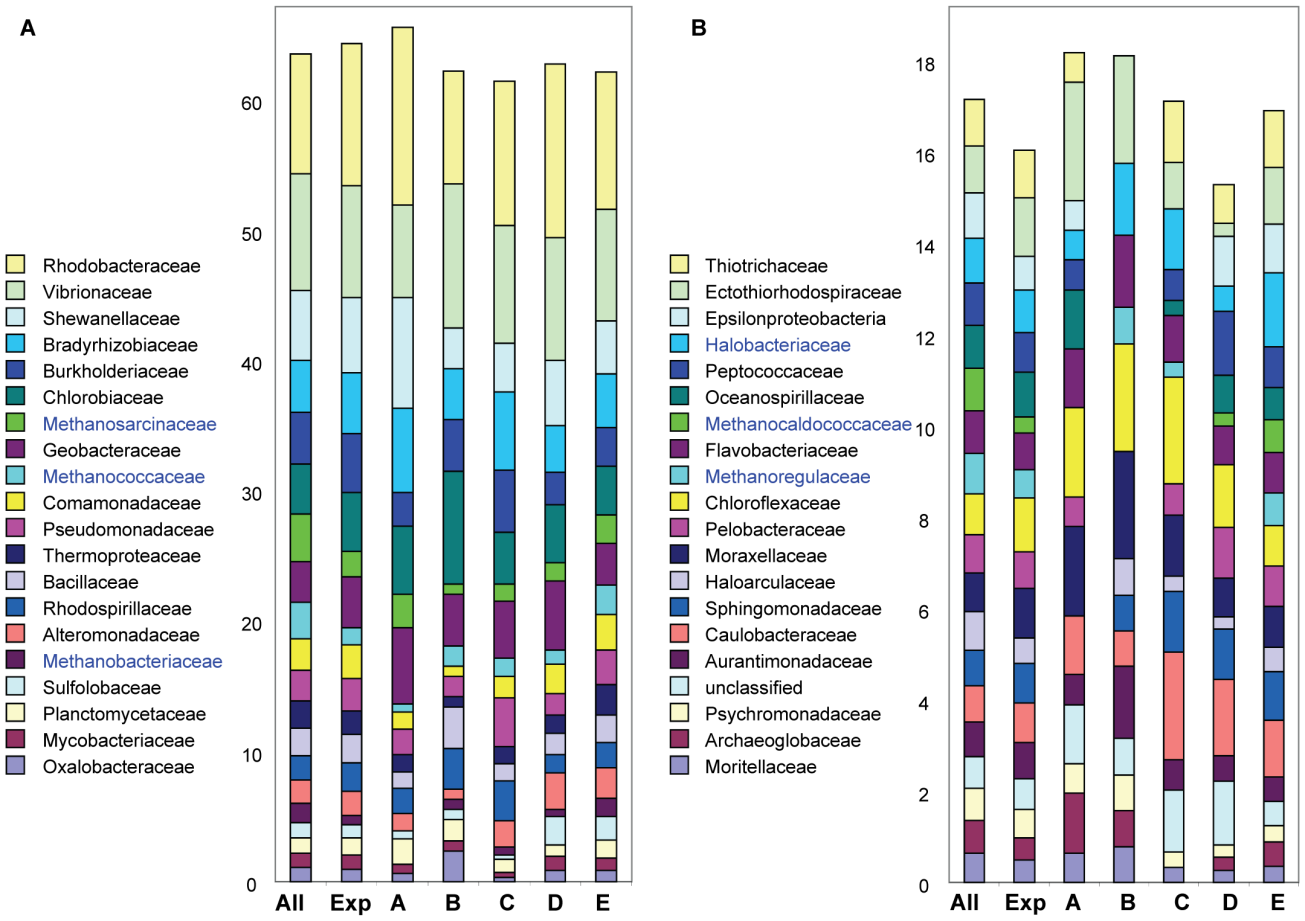
Phylogenetic analysis of differentially expressed microarray gene sets. For each cluster (corresponding to clusters A–E in Fig. 5), all genes upregulated over 2-fold relative to the seasonal freshwater baseline in at least 2 samples were selected (for cluster A, 160; B, 135; C, 301; D, 372; and E, 575 genes). The percentages of different prokaryotic families within each gene set are shown by different colors within the bar graphs. To the left is shown family representation of the entire probe set and of the probe set corresponding to expressed genes. Only families represented by a minimum of 15 probes are shown, these comprise 40 of the 96 total families. (A) The top 20 microbial families represented by >61% of the 2226 probe set; and (B) the next 20 microbial families represented by >16% of all probes. Euryarchaeota families with relatively high differential gene expression in cluster E are indicated in blue.

Consistent with our probe validation results, we analyzed the phylogenetic composition of the differentially expressed gene sets corresponding to clusters A through E (Fig. 5) at the family level (Fig. 9). For samples within each cluster we selected the probes that exhibited high signals relative to the freshwater baseline (the numbers of probes ranged from 135 to 578, depending on the cluster). Finally, we calculated the relative percentages of corresponding differentially expressed genes representing each microbial family. The relative representation of each family is also shown for the entire probe set, as well as for all probes producing signals above background (the 2076 probe set in Fig. 4). The composition of families represented in each cluster differed from the initial probe set and from one another. Although variability in the numbers of families represented by differentially-expressed genes in the different clusters was not large, there was a general trend toward higher diversity with increasing differential gene expression. Thus, cluster A contained 35 families and cluster B, 34 families with differentially expressed genes, while clusters C–E, showing more elevated levels of differential gene expression, contained 37, 39 and 40 families, respectively. Some families in the initial probe set were not detected in particular clusters. A striking example of this was the absence of methanogen families in cluster A. Of the five families of methanogens represented by the probe set, three were not detected in cluster A, and a fourth was reduced relative to its representation in the other clusters. In contrast, differential expression was detected from all five methanogen families in cluster E, and from four of the five families in clusters B, C and D (Fig. 9A). Interestingly, two other families from the top 40 representative families were not detected in cluster A, and one of these was Haloarcula, another member of the Euryarchaeota.

The highest similarity in phylogenetic composition was observed between cluster D and E gene sets (*R^2^* = 0.96), consistent with the sample clustering analysis described above (Fig. 5). In contrast to the other three clusters, D and E gene sets contained samples with relatively low differential expression of genes from Chloroflexaceae and Moraxellaceae. Distinguishing the two, however, was the relatively high differential expression of Euryarchaeota genes corresponding to several different families in cluster E (Fig. 9, shown in blue), and relatively low expression of Opitutaceae genes (data not shown). Finally, cluster B (August cluster) contained samples with relatively high differential expression of Chlorobiaceae, Oxalobacteraceae, and Aurantimonadaceae genes (Fig. 9). Taken together, ourdata indicated that gene expression changes relative to freshwater and seasons occurred in phylogenetically diverse microorganisms (Fig. 5, 9).

### Conclusions

In this study we employed a functional gene microarray probe set for detection of target genes and their closely related homologs in bacteria and archaea from environmental samples. This initial probe set was not intended to provide comprehensive coverage, since so little genome sequence information was available for CRCM microorganisms. Our aim, instead, was to test the efficacy of gene expression microarrays for detecting microbial responses to environmental change. Since many of the analyzed genes showed relatively high expression, coverage by the current probe set is likely to be representative of several important functional processes. Expansion of the probe set and additional improvements are planned for future work.

Application of multiple bioinformatic and experimental probe validation analyses consistently yielded 85–90% specificity for detection of designated targets, with 10–15% recognition of homologs from organisms within the same microbial family. Because of the likelihood that our probes hybridized to homologs of their target gene in at least some environmental samples, we limited our interpretations of trends in the data to those involving relatively large gene sets (e.g., Fig. 6, 7, 8). We have furthermore avoided conclusions relating functional processes to specific genera or species, and have instead analyzed gene expression profiles at higher taxonomic levels. If gene expression in the CRCM by members of a particular genus or species is of particular interest, additional analyses will need to be performed to provide exact identification. A more general interpretation of gene expression profiles, however, provided us with information about large-scale responses of prokaryotic assemblages during different seasons in the CRCM.

The strong seasonal shift observed in gene expression profiles between April and August is likely to be related to seasonal differences in nutrient inputs into the CRCM. In the Columbia River, as in other estuaries in this region [2], [33], river flows dominate nitrogen inputs into the estuary in winter and spring, while upwelling has a greater influence in summer and fall. Our gene expression data imply that the microbial community response to these nutrient inputs may also be different. Interestingly, the upwelling index for August 2007 was relatively low (data not shown), potentially corresponding to the relatively low levels of differential gene expression observed in the estuary, plume and adjacent coastal ocean relative to freshwater during that time. Given that differential gene expression in April was comparatively elevated, these observations may suggest that higher nutrient inputs into the estuary contribute to elevated levels of differential gene expression by bacteria and archaea relative to expression upriver, while lower nutrient inputs result in more similarity in gene expression throughout the system. Multi-year analyses to assess variation in gene expression in response to variability in composition, quantity and source of nutrient inputs will be necessary to test this hypothesis.

Taken together, our results suggest that river-derived nutrients, which build up over winter, have large effects on gene expression of microbial assemblages in the estuary, plume and adjacent coastal waters in the spring when temperature and light become favorable for growth. Both April, prior to the spring freshet, and August in late summer were productive by a number of criteria, including measurements of total RNA, chl *a*, and overall gene expression. The presence of abundant microbial populations in the estuary in April coincided with elevated differential gene expression relative to the end-member freshwater samples and involved large numbers of probes (370–500). Microbial biomass and gene expression was also relatively high in the river and plume in August, but for reasons discussed above related to lower nutrient inputs and perhaps also because phytoplankton were abundant during this time, little differential gene expression was observed. Our data also suggest that generally unfavorable conditions in the estuary (winter conditions in November, and record high river discharge in June) resulted in relatively lower microarray signals and limited elevation of gene expression (observed only for 160 genes) for most of the corresponding samples. Thus, our data support the hypothesis that during periods of high riverflow both estuarine nutrient gradients and estuarine microbial communities are strongly influenced by river discharge [32].

Our analysis of geochemical gradients indicated that no single factor was limiting for the development of productive microbial assemblages. Instead, microbial activity may have been determined by multiple combinations of environmental factors, such as described in a large body of literature discussing resource co-limitation in oceans (for review see [34]). Nevertheless, we were able to discern certain trends in differential gene expression related to combinations of light, location, salinity, nutrient concentrations, and phytoplankton biomass. Somewhat surprising was the fact that, within the estuary, salinity by itself did not appear to more strongly influence gene expression profiles. This may be a consequence of adaptation by microbial populations to constantly fluctuating salinities resulting from the dynamic mixing of seawater and freshwater.

The seasonal and inter-annual variability of external forcing (e.g., Fig. S5) and circulation conditions in the Columbia River are too high to allow an exhaustive characterization of microbial gene expression based on two years of field campaigns. Continuing campaigns (e.g., 2009–2010) will help, but our ultimate goal is the development of in-situ continuous microbial observations. Sensors to accomplish these types of observations are being developed at CMOP [62] and may additionally be supplemented with advanced instrumentation developed elsewhere [63]. Although the microarray profiles generated from this work are complex and their interpretation with respect to growth and activity of microbial populations in the CRCM will require ongoing analyses, our results indicate that environmental data and gene expression data may correspond in ways that facilitate our understanding of the drivers – physical, chemical, and microbial – of estuarine processes.

## Materials and Methods

### Collection of water samples

Water samples from the tidal freshwater, estuary, plume and adjacent coastal ocean were collected during four CMOP research cruises in August 2007, November 2007, April 2008, and June 2008, as described in detail in http://www.stccmop.org/research/cruise. Locations of the sampling stations are shown in the map in Fig. 1A. For each location, water was collected at the surface (1–3 m), and at a mid-depth ranging from 4 to 50 m depending on salinity, and, whenever possible, in association with a peak in chl *a* fluorescence. Samples were collected using 10-L Niskin bottles mounted to a CTD (conductivity-temperature-depth meter) rosette. All microorganisms present in the water column were collected by filtering water through Sterivex 0.22 µm filter units (Fisher Scientific, Waltham, MA). The volume of filtered water varied from 1 to 6 liters depending on rapidity of filter clogging (typically, 4–6 liters in the plume and coastal ocean, and 1–2 liters in the river), and was recorded for each sample. The filters were preserved in 2 mL of RNAlater reagent (Ambion, Austin, TX), frozen, and stored at -80°C.

### Environmental data acquisition

During sample collection, water temperature, salinity, and depth were recorded with a Sea-Bird 911+ CTD (conductivity-temperature-depth) profiler (Sea-Bird Electronics Inc., Bellevue, WA). Dissolved oxygen SBE43 (Sea-Bird) and chl *a* fluorescence (WetStar, WET Labs, Philomath, OR) sensors provided additional data. Aliquots of each water sample were used to obtain chemical and biological data, including nutrient concentrations, bacterial production, and chl *a* and pheophytin *a* fluorescence. Selected chemical data for the analyzed samples sets are shown in Fig. 1–2, while the entire suite of measurements is shown in Table S1. Daily values of river discharge at the Bonneville Dam, near Stevenson, Oregon (Fig. 1B) were acquired from the U.S. Geologic Survey's National Stream Water Quality Network (http://water.usgs.gov/nasqan/). Macronutrients (nitrate + nitrite – referred to hereafter as nitrate, ammonium, silicic acid and phosphate) were measured on a Lachat QuikChem 8000 Flow Injection Analysis system using standard colorimetric methods [64]. Bacterial production was measured as the rate of incorporation of L-[^3^H]leucine (50 nmol/L final concentration) using methods described elsewhere [39]. Dissolved organic carbon was measured by filtering 20 mL of water through a GFF filter (ø 25 mm, Whatman, Piscataway, NJ). The filters were analyzed by HPL analytical services as described [65] using a TOC-5000 total organic carbon analyzer (Shimadzu, Columbia, MD). Particulate organic carbon (POC) was measured by filtering 100–300 mL of water onto a pre-combusted (12 hours at 500°C) GFF filter (ø 25 mm, Whatman) to collect suspended particulate matter for elemental analysis. The POC content on the acid-fumed filters was determined using a Carlo Erba NA-1500 Elemental Analyzer (Thermo Fisher Scientific, Waltham, MA), configured and operated as described [66].

### Total RNA isolation from filter samples

For each filtered water sample, total RNA was isolated from all microorganisms, both prokaryotic and eukaryotic, that were retained on 0.2 µm filters. The RNA isolation protocol described in Griffiths *et al* [16] was modified in the following manner. Each Sterivex filter was removed from the holder, cut longitudinally and placed into 2 ml screw-top eppendorf tubes containing CTAB extraction buffer (5% CTAB, 0.8 M NaCl in 0.1 M phosphate buffer, pH 8.0) and 0.5 mL zirconia/silica beads (1∶1 mixture of 0.1 and 1 mm, BioSpec Products, Bartlesville, OK). Bead beating for microbial cell disruption was performed for 1 min using a FastPrep FP120 machine (Thermo Fisher Scientific, Waltham, MA). The filter strips were re-extracted 3 times to maximize RNA yield, after which the extracts were pooled together and supplemented with an equal volume of a 1∶1 mixture of phenol:chloroform, and 0.1 volume each of 10% SDS, and 10% sodium lauryl sarcosine. Sample extracts were incubated for 20 min at room temperature with intense shaking (80 rpm), followed by standard phenol:chloroform extraction and isopropanol precipitation. The extracted RNA was treated with RNase-free DNase (Qiagen, Germantown, MD), and then purified using the RNeasy Kit (Qiagen) as recommended by the manufacturer. The RNA content was measured spectrophotometrically with a Nanodrop 3300 instrument (Thermo Fisher Scientific), and RNA quality was evaluated by capillary electrophoresis using an Agilent 2100 Bioanalyzer (Agilent Technologies, Palo Alto, CA). Prominent rRNA peaks (both prokaryotic and eukaryotic) were observed in all samples. RNA concentrations were normalized per liter of filtered water to generate the RNA values shown in Fig. 3C. We used several sets of replicate samples to analyze the reproducibility of our RNA isolation procedure and the variability in total RNA content introduced by sample handling during water collection, filtering, and on-board storage. In all cases, RNA concentrations calculated for independently processed replicate samples had low coefficients of variation (4 to 14%, data not shown). Thus, the normalized RNA concentrations were used as a measurement of total living microbial biomass in the samples.

### Preparation of fluorescently labeled cDNA targets for microarray hybridization

Total RNA samples were converted into cDNA using the Superscript III Reverse Transcriptase Kit (Invitrogen, Carlsbad, CA) with random hexamer primers. RNA was removed with RNase H according to the manufacturer's protocol. The cDNA samples were purified using QIAQuick Nucleotide Removal Kit (Qiagen), and fluorescently labeled using LabelIT uArray Cy5 Kit (Mirus Bio, Madison WI) as described by the manufacturer. The Cy5-labeled cDNA samples were purified using Illustra ProbeQuant G-50 Micro Columns (GE Healthcare, Piscataway, NJ), dried in a SpeedVac centrifuge and applied for microarray hybridization.

### Subtractive hybridization assays

Partial depletion of rRNA from total RNA was performed using MICROBExpress™ Bacterial mRNA Enrichment Kit (Ambion, Austin, TX) as recommended by the manufacturer. A pooled sample of total RNA was divided into two aliquots, one of which was subjected to rRNA subtractive hybridization and removal using universal rRNA-specific primers and magnetic beads. Target cDNA samples were prepared independently for both untreated and partially depleted aliquots and equal amounts of each target preparation were hybridized to replicate microarrays. Results indicated that the relative rRNA content of this aliquot decreased to approximately 80% (from >95%), as estimated by capillary electrophoresis (data not shown).

### Selection of genes of interest and microarray design

Genes of interest were selected using the Integrated Microbial Genomes (IMG) 2.5 system from DOE Joint Genome Institute [28]. Approximately 300 sequenced genomes from environmental bacteria and archaea (251 and 48, respectively) were used for keyword-based selection of genes from those genomes with well-defined functional annotation related to carbon and nitrogen metabolism (which made up 48% and 27% of the selected genes, respectively). The remaining 25% of selected genes encoded well-characterized enzymes of central metabolism (housekeeping functions) and a small number (37) of light perception genes.

Oligonucleotide probes were designed from the list of genes of interest using Probe Weaver software (CombiMatrix Corporation, Mukilteo, WA). The recommended standard settings were used for selection of 35- to 45-nt oligomers in the sense orientation, with the melting temperature of target:probe hybrids varying from 70°C to 75°C. The software was used to select probes based on their specificity within the submitted gene set. Thus, to prevent cross-hybridization of the selected probes with rRNA, 146 and 882 rRNA genes from eukaryotic and prokaryotic microorganisms, respectively, were added to the submitted gene list. These rRNA sequences were not used to design probes, but rather to filter the selected probes against cross-hybridization. After completion of the probe design, the probe sets were synthesized in situ on the surface of CombiMatrix CustomArray™ 12K and 4X2K oligonucleotide microarrays (described in www.combimatrix.com).

### Microarray hybridization and re-use

Hybridization of CustomArray™ microarrays was performed as recommended by the manufacturer (http://www.combimatrix.com/docs/PTL005_00_4x2K_Hyb_Imaging.pdf), using the single-color experimental scheme. The microarrays were hybridized at 46°C for 16 hours, using Cy5-labeled cDNA targets, 3–4 µg per microarray for the 12K format, or 1.5–2 µg per sector for the 4X2K format. Hybridization images were obtained using a ScanArray 4000 fluorescent scanner (PerkinElmer, Waltham, MA) at 5 micron resolution, and quantified with Microarray Imager software (CombiMatrix). For microarray re-use, the labeled targets were chemically denatured and removed from microarray probes as described in the CombiMatrix stripping protocol (http://www.combimatrix.com/docs/PTL002_01_4x2K_StrippingReHyb.pdf). The quality of target removal was evaluated by scanning at 5 micron resolution. In total, each microarray was used four times, as recommended by the manufacturer.

### Microarray data analysis and deposition

Data normalization based on the quantile algorithm was done using the Probe Weaver software from CombiMatrix. The background signal value was calculated as the mean intensity of the lowest 5% of all signals. Genes were selected as significantly expressed if the corresponding probes produced normalized signal intensities exceeding the background value plus 3X standard deviation in at least 2 different samples. Gene expression ratios for each seasonal sample set were calculated versus corresponding freshwater river baselines. For each gene represented on the microarrays, the baseline was calculated as the average of the normalized signal intensities in two independent samples collected at the Beaver Army Terminal (River Mile 53, Fig. 1A, the corresponding samples are shown in bold in Table S1). Ratios of differential gene expression were then calculated for each gene in the remaining 56 samples as log_2_ (signal intensity of the gene in the sample) minus log_2_ (intensity of the gene in the corresponding seasonal river baseline). Finally, to select for statistically significant changes, genes were designated as ‘differentially expressed’ if the corresponding ratios varied by at least 2-fold in at least 2 out of 56 samples (consistent with recommended criteria [67], [68]). Scatter plot analysis of duplicate hybridizations, calculation of pair-wise correlation coefficients among samples, and ANOVA were done using NCI-supported software BRB-ArrayTools (http://linus.nci.nih.gov/pilot/index.html). Clustering and self-organizing map (SOM) analyses were performed using Cluster and TreeView software (http://rana.lbl.gov/eisen/ [69]). The data discussed in this publication have been deposited in NCBI's Gene Expression Omnibus [70], and are accessible through GEO Series accession number GSE18303 (http://www.ncbi.nlm.nih.gov/geo/query/acc.cgi?acc=GSE18303).

## Supporting Information

Figure S1 Validation of microarray probe specificity with Bacillus subtilis RNA. Total RNA was isolated from laboratory cultures of Bacillus subtilis, converted to fluorescently labeled cDNA, and hybridized in duplicate to microarrays. (A) Representative plot of the raw signal intensity data sorted by organism annotations; (B) raw signal intensity data for a subset of probes corresponding to Bacilli/Bacillales probes only.
(0.18 MB TIF)

Figure S2 Experimental variation and reproducibility of microarray data. Microarray data were analyzed using scatter plots of replicate experiments. Each data point on a scatter plot represents an individual probe. X and Y values are the normalized signal intensities (log scale) in the first and second hybridizations, respectively. The angled lines show the two-fold cut-offs for the ratios between X and Y values. Data points located outside of the lines show significant (over 2-fold) difference between values obtained in the first and second hybridizations, and they are indicated with closed circles. The data points located within the cut-off lines are indicated with open circles.
(0.82 MB TIF)

Figure S3 Pair-wise correlation (Spearman rank) coefficients calculated for four sampling seasons. Within each season,microarray profiles for each sample were compared to those for the corresponding seasonal freshwater end-member (collected at Beaver Army Dock, Fig. 1A). The resulting correlation coefficients were plotted according to water salinities. Black and gray squares represent ocean and river samples, respectively. Trend lines were generated in Excel.
(0.12 MB TIF)

Figure S4 Multidimensional scaling (MDS) plots of maximum variability among samples. MDS plots were constructed using Primer6 software. Samples are displayed according to sampling time (A, April; J, June; AG, August; N, November).
(0.31 MB TIF)

Figure S5 Seasonal and inter-annual variability of Columbia River discharges. Recent years (1997 and 2001) with extreme discharge levels are shown for context relative to the years of CMOP campaigns to date (2007–2009; campaign periods marked at the bottom of the graph). In light gray, discharges for all other years since 1997 are shown. Inter-annual variations are most marked in winter and during spring freshets (May-June), but are present throughout the year. Q, river discharge in m3s-1.
(0.20 MB TIF)

Table S1 Corresponding physical, chemical and biological characteristics of the seasonal sample sets analyzed by microarray analysis. Location codes: number shows distance from the coast in km; CR, Columbia River transect in the plume and coastal ocean; NH, Newport Hydroline transect in the coastal ocean at Newport, Oregon; AST and HAM, CRE locations near Astoria (river mile 7–9) and Hammond (river mile 5), respectively; TID, CRE locations in the tidal basin (river mile 22–23); BA, river location at Beaver Army Dock (river mile 53) near Quincy, Oregon; UP, river at mile 74. River baseline samples are shown in bold. Leucine incorp., leucine incorporation by heterotrophic plankton.
(0.64 MB TIF)
