# Supplementary figures and images for: Seasonal Changes in Bacterial and Archaeal Gene Expression Patterns across Salinity Gradients in the Columbia River Coastal Margin
Source: PLoS One. 2010 Oct 13;5(10):e13312. doi: 10.1371/journal.pone.0013312 (PMC2954162; doi:10.1371/journal.pone.0013312)

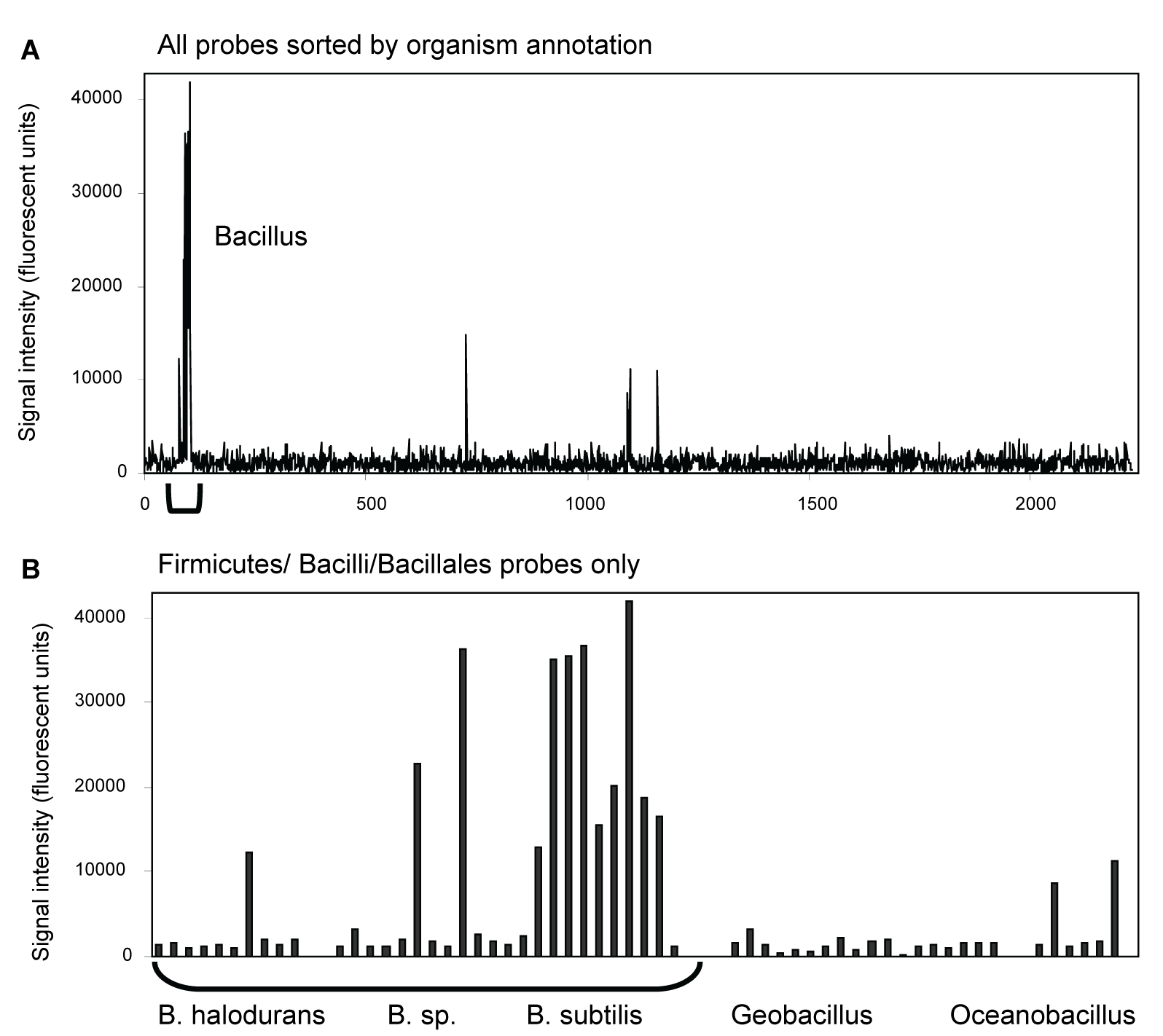

Supplement: Figure S1 — Validation of microarray probe specificity with Bacillus subtilis RNA. Total RNA was isolated from laboratory cultures of Bacillus subtilis, converted to fluorescently labeled cDNA, and hybridized in duplicate to microarrays. (A) Representative plot of the raw signal intensity data sorted by organism annotations; (B) raw signal intensity data for a subset of probes corresponding to Bacilli/Bacillales probes only. (0.18 MB TIF) [file pone.0013312.s001.tif]

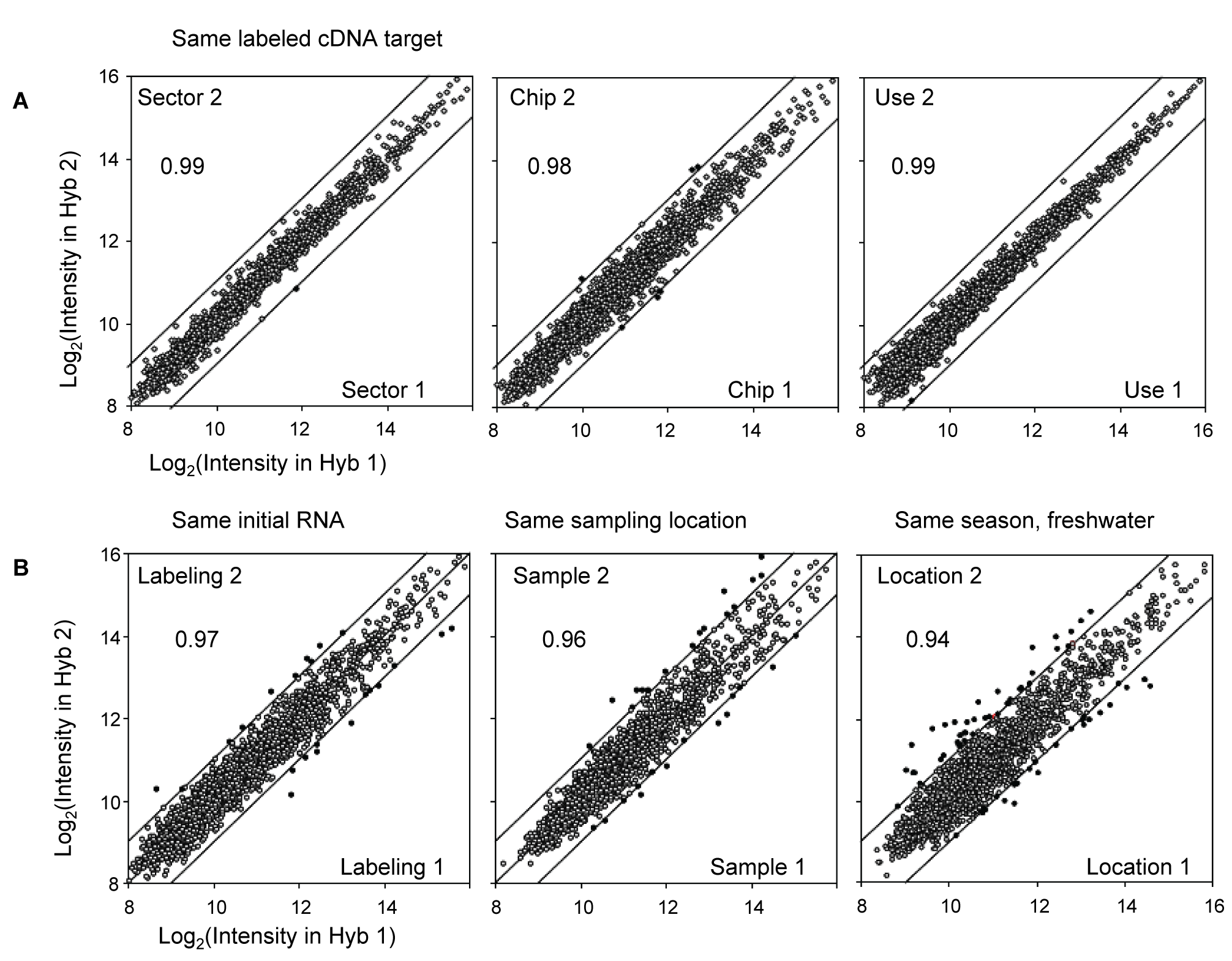

Supplement: Figure S2 — Experimental variation and reproducibility of microarray data. Microarray data were analyzed using scatter plots of replicate experiments. Each data point on a scatter plot represents an individual probe. X and Y values are the normalized signal intensities (log scale) in the first and second hybridizations, respectively. The angled lines show the two-fold cut-offs for the ratios between X and Y values. Data points located outside of the lines show significant (over 2-fold) difference between values obtained in the first and second hybridizations, and they are indicated with closed circles. The data points located within the cut-off lines are indicated with open circles. (0.82 MB TIF) [file pone.0013312.s002.tif]

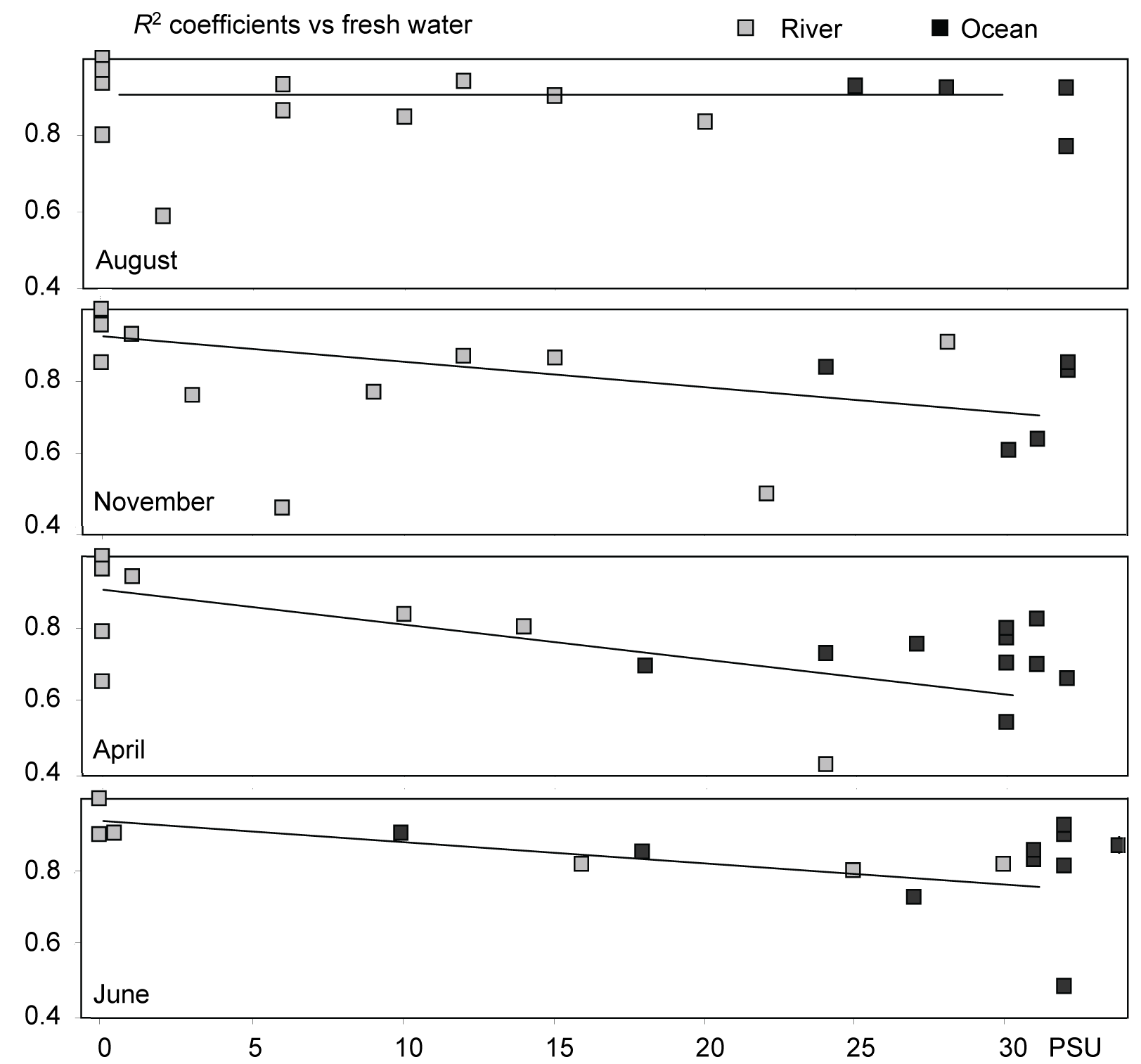

Supplement: Figure S3 — Pair-wise correlation (Spearman rank) coefficients calculated for four sampling seasons. Within each season,microarray profiles for each sample were compared to those for the corresponding seasonal freshwater end-member (collected at Beaver Army Dock, Fig. 1A). The resulting correlation coefficients were plotted according to water salinities. Black and gray squares represent ocean and river samples, respectively. Trend lines were generated in Excel. (0.12 MB TIF) [file pone.0013312.s003.tif]

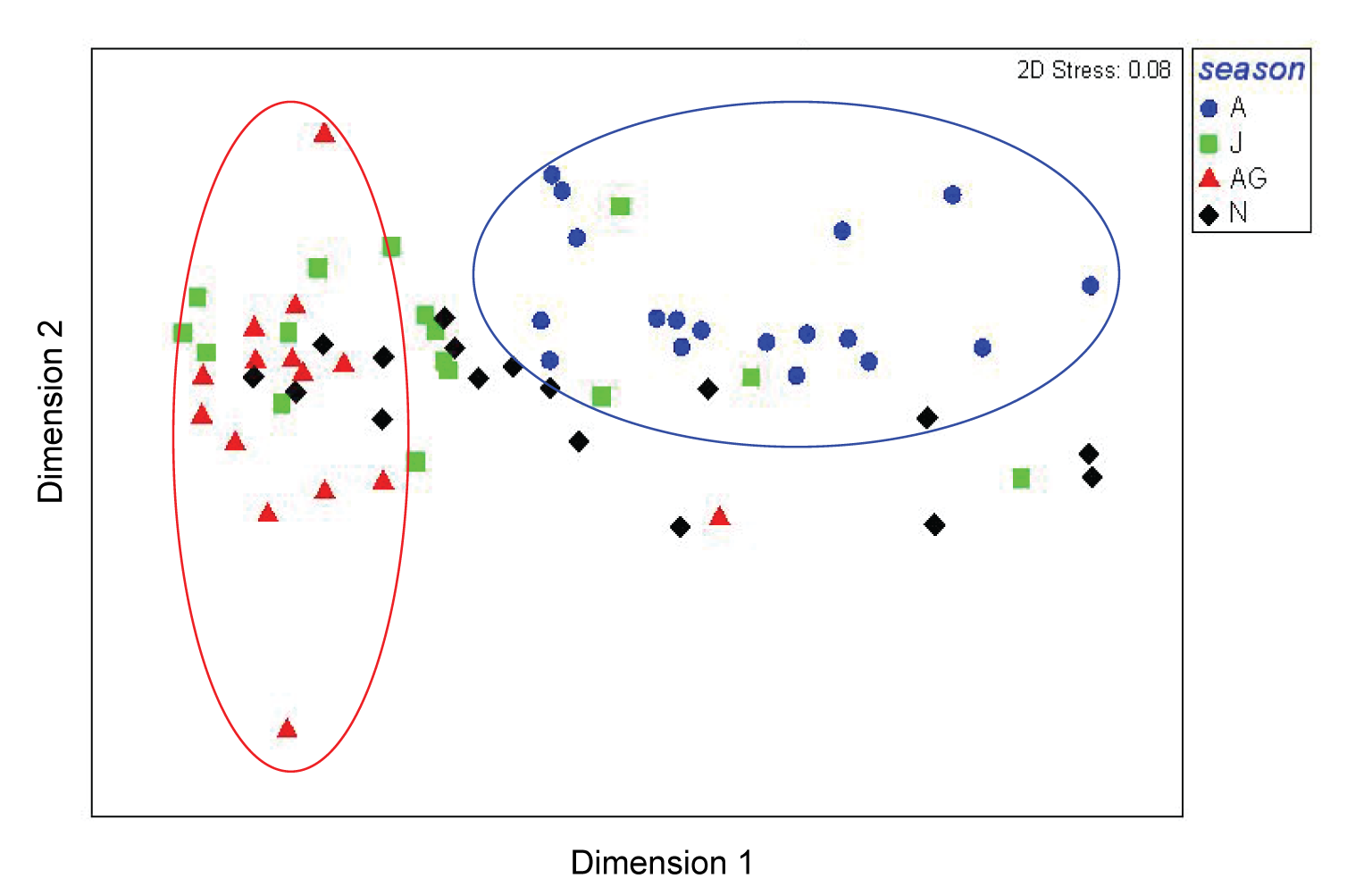

Supplement: Figure S4 — Multidimensional scaling (MDS) plots of maximum variability among samples. MDS plots were constructed using Primer6 software. Samples are displayed according to sampling time (A, April; J, June; AG, August; N, November). (0.31 MB TIF) [file pone.0013312.s004.tif]

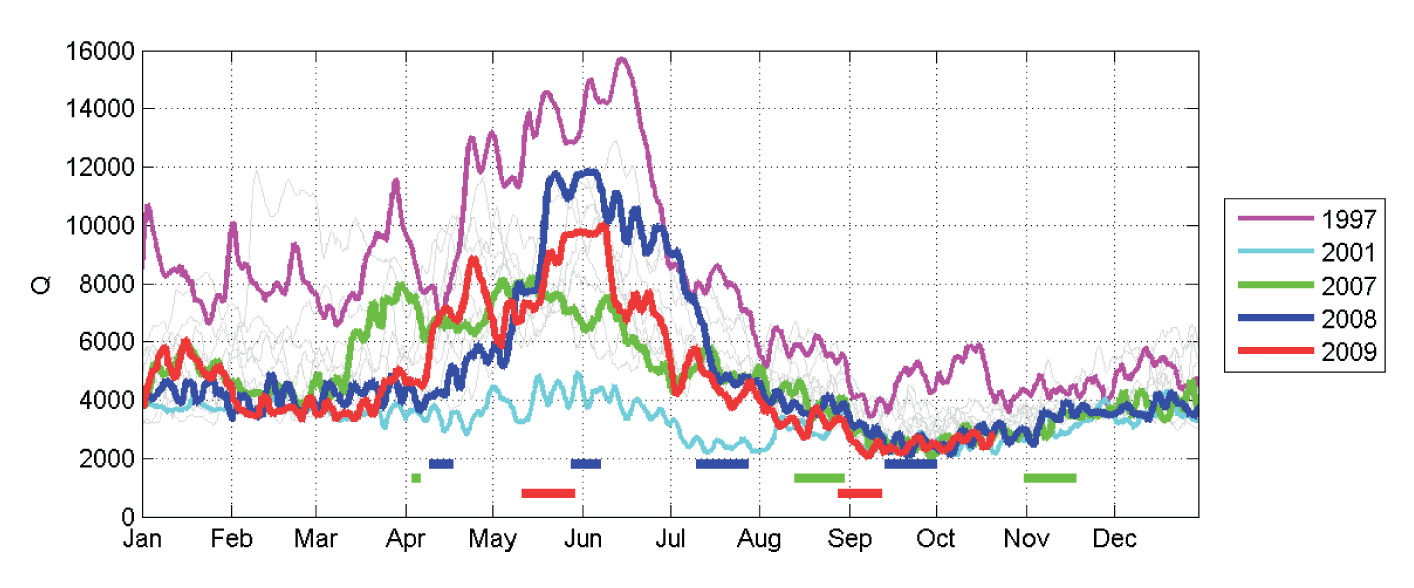

Supplement: Figure S5 — Seasonal and inter-annual variability of Columbia River discharges. Recent years (1997 and 2001) with extreme discharge levels are shown for context relative to the years of CMOP campaigns to date (2007–2009; campaign periods marked at the bottom of the graph). In light gray, discharges for all other years since 1997 are shown. Inter-annual variations are most marked in winter and during spring freshets (May-June), but are present throughout the year. Q, river discharge in m3s-1. (0.20 MB TIF) [file pone.0013312.s005.tif]

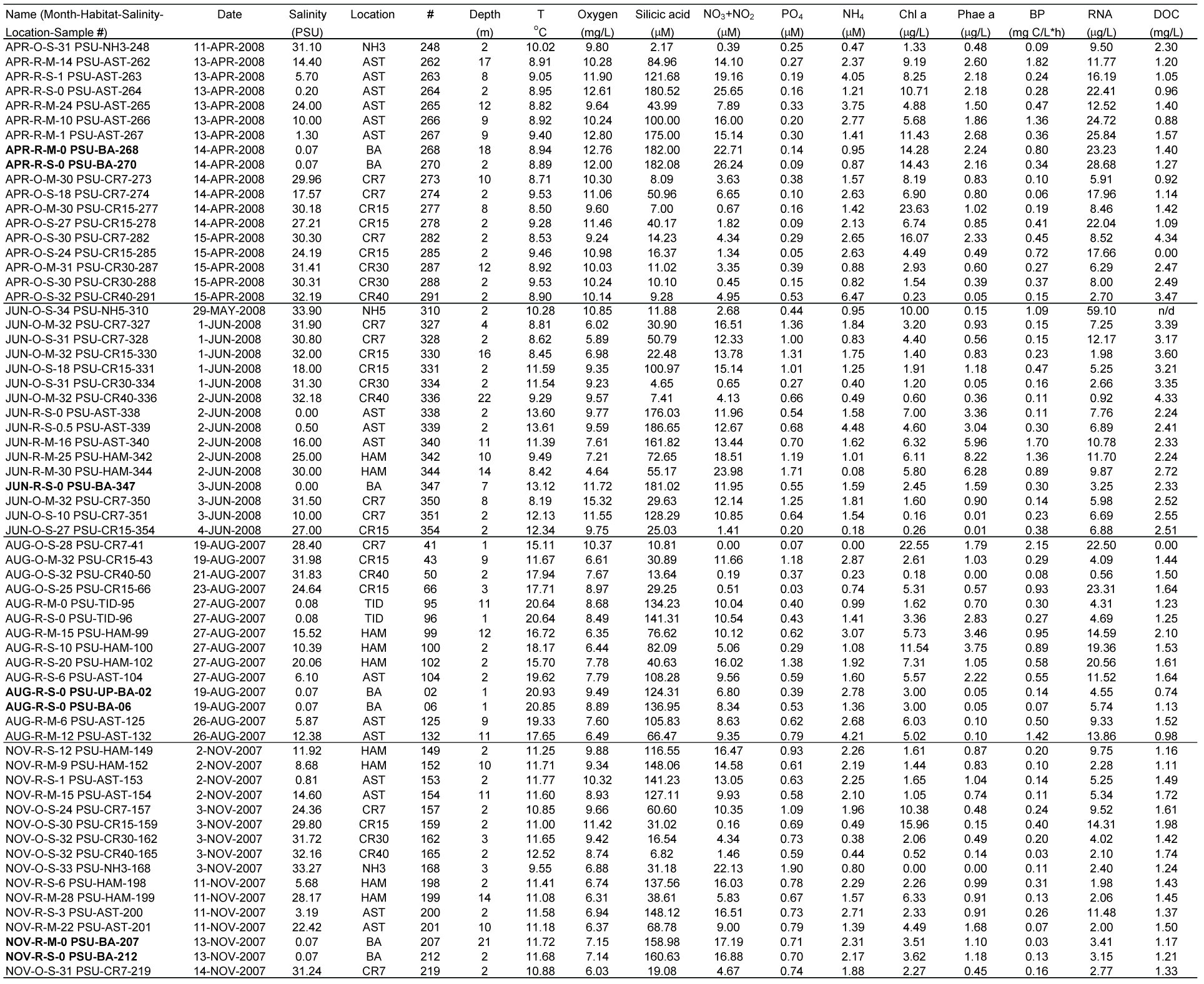

Supplement: Table S1 — Corresponding physical, chemical and biological characteristics of the seasonal sample sets analyzed by microarray analysis. Location codes: number shows distance from the coast in km; CR, Columbia River transect in the plume and coastal ocean; NH, Newport Hydroline transect in the coastal ocean at Newport, Oregon; AST and HAM, CRE locations near Astoria (river mile 7–9) and Hammond (river mile 5), respectively; TID, CRE locations in the tidal basin (river mile 22–23); BA, river location at Beaver Army Dock (river mile 53) near Quincy, Oregon; UP, river at mile 74. River baseline samples are shown in bold. Leucine incorp., leucine incorporation by heterotrophic plankton. (0.64 MB TIF) [file pone.0013312.s006.tif]
